# Universally Sloppy Parameter Sensitivities in Systems Biology Models

**DOI:** 10.1371/journal.pcbi.0030189

**Published:** 2007-10-05

**Authors:** Ryan N Gutenkunst, Joshua J Waterfall, Fergal P Casey, Kevin S Brown, Christopher R Myers, James P Sethna

**Affiliations:** 1 Laboratory of Atomic and Solid State Physics, Cornell University, Ithaca, New York, United States of America; 2 Department of Molecular Biology and Genetics, Cornell University, Ithaca, New York, United States of America; 3 UCD Conway Institute of Biomolecular and Biomedical Research, University College Dublin, Belfield, Ireland; 4 Department of Molecular and Cellular Biology, Harvard University, Cambridge, Massachusetts, United States of America; 5 Cornell Theory Center, Cornell University, Ithaca, New York, United States of America; Lawrence Berkeley National Laboratory, United States of America

## Abstract

Quantitative computational models play an increasingly important role in modern biology. Such models typically involve many free parameters, and assigning their values is often a substantial obstacle to model development. Directly measuring in vivo biochemical parameters is difficult, and collectively fitting them to other experimental data often yields large parameter uncertainties. Nevertheless, in earlier work we showed in a growth-factor-signaling model that collective fitting could yield well-constrained predictions, even when it left individual parameters very poorly constrained. We also showed that the model had a “sloppy” spectrum of parameter sensitivities, with eigenvalues roughly evenly distributed over many decades. Here we use a collection of models from the literature to test whether such sloppy spectra are common in systems biology. Strikingly, we find that every model we examine has a sloppy spectrum of sensitivities. We also test several consequences of this sloppiness for building predictive models. In particular, sloppiness suggests that collective fits to even large amounts of ideal time-series data will often leave many parameters poorly constrained. Tests over our model collection are consistent with this suggestion. This difficulty with collective fits may seem to argue for direct parameter measurements, but sloppiness also implies that such measurements must be formidably precise and complete to usefully constrain many model predictions. We confirm this implication in our growth-factor-signaling model. Our results suggest that sloppy sensitivity spectra are universal in systems biology models. The prevalence of sloppiness highlights the power of collective fits and suggests that modelers should focus on predictions rather than on parameters.

## Author Summary

Dynamic systems biology models typically involve many kinetic parameters, the quantitative determination of which has been a serious obstacle to using these models. Previous work showed for a particular model that useful predictions could be extracted from a fit long before the experimental data constrained the parameters, even to within orders of magnitude. This was attributed to a “sloppy” pattern in the model's parameter sensitivities; the sensitivity eigenvalues were roughly evenly spaced over many decades. Consequently, the model behavior depended effectively on only a few “stiff” parameter combinations. Here we study the converse problem, showing that direct parameter measurements are very inefficient at constraining the model's behavior. To yield effective predictions, such measurements must be very precise and complete; even a single imprecise parameter often destroys predictivity. We also show here that the characteristic sloppy eigenvalue pattern is reproduced in 16 other diverse models from the systems biology literature. The apparent universality of sloppiness suggests that predictions from most models will be very fragile to single uncertain parameters and that collective parameters fits can often yield tight predictions with loose parameters. Together these results argue that focusing on parameter values may be a very inefficient route to useful models.

## Introduction

Dynamic computational models are powerful tools for developing and testing hypotheses about complex biological systems [1–3]. It has even been suggested that such models will soon replace databases as the primary means for exchanging biological knowledge [4]. A major challenge with such models, however, is that they often possess tens or even hundreds of free parameters whose values can significantly affect model behavior [5,6]. While high-throughput methods for discovering interactions are well-developed [7], high-throughput methods for measuring biochemical parameters remain limited [8]. Furthermore, using values measured in vitro in an in vivo application may introduce substantial inaccuracies [9,10]. On the other hand, collectively fitting parameters [11,12] by optimizing the agreement between the model and available data often yields large parameter uncertainties [13–15]. In approaches typically more focused on steady-state distributions of fluxes in metabolic networks, metabolic control analysis has been used to quantify the sensitivity of model behavior with respect to parameter variation [16], and flux-balance analysis and related techniques have probed the robustness of metabolic networks [17,18].

One way to cope with the dearth of reliable parameter values is to focus on predictions that are manifestly parameter-independent [19], but these are mostly qualitative. An alternative is not to forsake quantitative predictions, but to accompany them with well-founded uncertainty estimates based on an ensemble of parameter sets statistically drawn from all sets consistent with the available data [20]. (Uncertainties in the model structure itself may be important in some cases. Here we focus on parameter uncertainties, as they are often important on their own.) Brown et al. took this approach in developing a computational model of the well-studied growth-factor-signaling network in PC12 cells [21]. They collectively fit their model's 48 parameters to 68 data points from 14 cell-biology experiments (mostly Western blots). After the fit, all 48 parameters had large uncertainties; their 95% confidence intervals each spanned more than a factor of 50. Surprisingly, while fitting this modest amount of data did not tightly constrain any single parameter value, it did enable usefully tight quantitative predictions of behavior under interventions, some of which were verified experimentally.

In calculating their uncertainties, Brown et al. found that the quantitative behavior of their model was much more sensitive to changes in certain combinations of parameters than others. Moreover, the sensitivity eigenvalues were approximately equally spaced in their logarithm, a pattern deemed “sloppy.” Such sloppy sensitivities were subsequently seen in other multiparameter fitting problems, from interatomic potentials [22] to sums of exponentials [23]. The fact that sloppiness arises in such disparate contexts suggests that it may be a universal property of nonlinear multiparameter models. (Here the term “universal” has a technical meaning from statistical physics, denoting a shared common property with a deep underlying cause; see [23]. Universality in this sense does not imply that all models must necessarily share the property.)

In this work, we begin by empirically testing 17 systems biology models from the literature, examining the sensitivity of their behavior to parameter changes. Strikingly, we find that Brown et al.'s model is not unique in its sloppiness; every model we examine exhibits a sloppy parameter sensitivity spectrum. (Thus, in the models we've examined, sloppiness is also universal in the common English sense of ubiquity.) We then study the implications of sloppiness for constraining parameters and predictions. We argue that obtaining precise parameter values from collective fits will remain difficult even with extensive time-series data, because the behavior of a sloppy model is very insensitive to many parameter combinations. We also argue that, to usefully constrain model predictions, direct parameter measurements must be both very precise and complete, because sloppy models are also conversely very sensitive to some parameter combinations. Tests over our collection of models support the first prediction, and detailed analysis of the model of Brown et al. supports the second contention.

Sloppiness, while not unique to biology, is particularly relevant to biology, because the collective behavior of most biological systems is much easier to measure in vivo than the values of individual parameters. Much work has focused on optimizing experimental design to best constrain model parameters with collective fits [24–26]. We argue against this focus on parameter values, particularly when our understanding of a system is tentative and incomplete. Concrete predictions can be extracted from models long before their parameters are even roughly known [21], and, when a system is not already well-understood, it can be more profitable to design experiments to directly improve predictions of interesting system behavior [27] rather than to improve estimates of parameters.

## Results

### Systems Biology Models Have Sloppy Sensitivity Spectra

Our collection of 17 systems biology models [2,21,25,28–41] was drawn primarily from the BioModels database [42], an online repository of models encoded in the Systems Biology Markup Language (SBML) [43]. The collected models encompass a diverse range of biological systems, including circadian rhythm, metabolism, and signaling. All the models are formulated as systems of ordinary differential equations, and they range from having about ten to more than 200 parameters. In most cases, these parameters were not systematically fit or measured in the original publication.

We quantified the change in model behavior as parameters *θ* varied from their published values *θ* ^*^ by the average squared change in molecular species time courses:

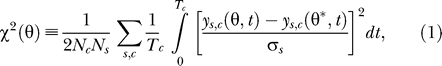
a kind of continuous least-squares fit of parameters *θ* to data simulated from published parameters *θ* ^*^. Here *y_s_*_,*c*_(*θ*,*t*) is the time course of molecular species *s* given parameters *θ* in condition *c*, and *T_c_* is the “measurement” time for that condition. We took the species normalization *σ_s_* to be equal to the maximum value of species *s* across the conditions considered; other consistent normalizations yield the same qualitative conclusions.

For each model, the sum in Equation 1 runs over all molecular species in the model and (except where infeasible) over all experimental conditions considered in the corresponding paper for each model—an attempt to neutrally measure system behavior under conditions deemed significant by the original authors. (The total number of conditions and species are denoted by *N_c_* and *N_s_*, respectively.) SBML files and SloppyCell [44] scripts for all models and conditions are available in Dataset S1.

**Figure 1:**
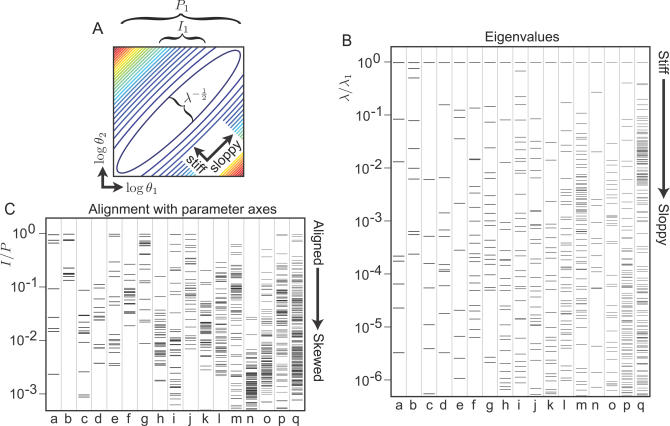
Parameter Sensitivity Spectra The quantities we calculate from *H* ^χ^2^^ are illustrated in (A), while (B) and (C) show that all the models we examined have sloppy sensitivity spectra. (A) Analyzing *H* ^χ^2^^ corresponds to approximating the surfaces of constant model behavior change (constant *χ* ^2^) as ellipsoids. The width of each principal axis is proportional to one over the square root of the corresponding eigenvalue. The inner ellipsoid's projection onto and intersection with the *θ*_1_ axis are denoted by *P*_1_ and *I_1_*, respectively. (B) Plotted are the eigenvalue spectra of *H* ^χ^2^^ for our collection of systems biology models. The many decades generally spanned indicate that the ellipses have a very large aspect ratio. (The spectra have each been normalized by their largest eigenvalue. Not all values are visible for all models.) (C) Plotted is the spectrum of *I* / *P* for each parameter in each model in our collection. Generally very few parameters have *I* / *P* ≈ 1, suggesting that the ellipses are skewed from the bare parameter axes. (Not all values are visible for all models.) The models are plotted in order of increasing number of free parameters and are: (a) eukaryotic cell cycle [28], (b) *Xenopus* egg cell cycle [29], (c) eukaryotic mitosis [30], (d) generic circadian rhythm [31], (e) nicotinic acetylcholine intra-receptor dynamics [32], (f) generic kinase cascade [33], (g) *Xenopus* Wnt signaling [34], (h) *Drosophila* circadian rhythm [35], (i) rat growth-factor signaling [21], (j) *Drosophila* segment polarity [36], (k) *Drosophila* circadian rhythm [37], (l) *Arabidopsis* circadian rhythm [2], (m) in silico regulatory network [25], (n) human purine metabolism [38], (o) Escherichia coli carbon metabolism [39], (p) budding yeast cell cycle [40], (q) rat growth-factor signaling [41].

To analyze each model's sensitivity to parameter variation, we considered the Hessian matrix corresponding to *χ* ^2^:

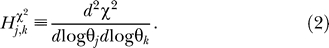
We took derivatives with respect to log *θ* to consider relative changes in parameter values, because biochemical parameters can have different units and widely varying scales. Analyzing *H* ^χ^2^^ corresponds to approximating the surfaces of constant model behavior deviation (as quantified by *χ* ^2^) to be *N_p_*-dimensional ellipsoids, where *N_p_* is the number of parameters in the model. Figure 1A schematically illustrates these ellipsoids and some of their characteristics. (Details of calculating *H* ^χ^2^^ and related quantities are found in Methods. Dataset S1 includes *H* ^χ^2^^ for each model.)

The principal axes of the ellipsoids are the eigenvectors of *H* ^χ^2^^, and the width of the ellipsoids along each principal axis is proportional to one over the square root of the corresponding eigenvalue. The narrowest axes are called “stiff,” and the broadest axes “sloppy” [20]. The eigenvalue spectra for the models in our collection are shown in Figure 1B (each normalized by its largest eigenvalue). In every case, the eigenvalues span many decades. All but one span more than 10^6^, indicating that the sloppiest axes of the ellipsoids illustrated in Figure 1A are generally more than 1,000 times as long as the stiffest axes. In each spectrum the eigenvalues are also approximately evenly spaced in their logarithm; there is no well-defined cutoff between “important” and “unimportant” parameter combinations.

The Hessian matrix is a local quadratic approximation to the generally nonlinear *χ* ^2^ function. Principal component analysis of extensive Monte Carlo runs in the Brown et al. model, however, indicates that the sloppiness revealed by *H* ^χ^2^^ is indicative of full nonlinear *χ* ^2^ function [20].

Along with their relative widths, the degree to which the principal axes of the ellipsoids are aligned to the bare parameter axes is also important. We estimated this by comparing the ellipsoids' intersections *I_i_* with each bare parameter axis *i* and projections *P_i_* onto each bare parameter axis *i*. If *I_i_* / *P_i_* = 1, then one of the principal axes of the ellipsoids lies along bare parameter direction *i*. Figure 1C plots the *I* / *P* spectrum for each model. In general, very few axes have *I* / *P* ≈ 1; the ellipses are skewed from single parameter directions.

Naively, one might expect the stiff eigenvectors to embody the most important parameters and the sloppy directions to embody parameter correlations that might suggest removable degrees of freedom, simplifying the model. Empirically, we have found that the eigenvectors often tend to involve significant components of many different parameters; plots of the four stiffest eigenvectors for each model are in Text S1. This is understandable theoretically; the nearly degenerate sloppy eigenvectors should mix, and the stiff eigenvectors can include arbitrary admixtures of unimportant directions to a given important parameter combination. (Indeed, in analogous random-matrix theories, the eigenvectors are known to be uncorrelated random vectors [45].) While the relatively random eigenvectors studied here may not be useful in guiding model reduction, more direct explorations of parameter correlations have yielded interesting correlated parameter clusters [46].

These characteristic parameter sensitivities that evenly span many decades and are skewed from bare parameter axes define a “sloppy” model [20]. Figure 1B and 1C shows that every model we have examined has a sloppy sensitivity spectrum. Next we discuss some broad questions about the relation between model predictions, collective fits, and parameter measurements and see how the sloppy properties of these models may suggest answers.

### Consequences of Sloppiness

**Figure 2:**
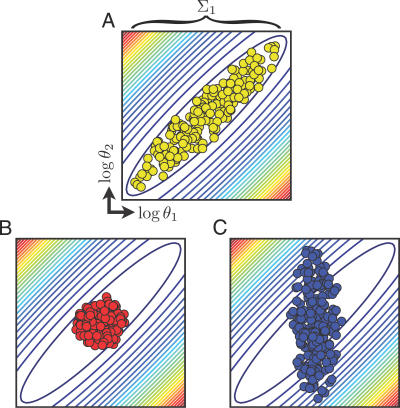
Sloppiness and Uncertainties As in Figure 1A, the contours represent surfaces of constant model behavior deviation. The clouds of points represent parameter set ensembles. (A) Collective fitting of model parameters naturally constrains the parameter set ensemble along stiff directions and allows it to expand along sloppy directions. The resulting ensemble may be very large, yet encompass little variation in model behavior, yielding small prediction uncertainties despite large parameter uncertainties. (Σ_1_ denotes the 95% confidence for the value of *θ*_1_.) (B) If all parameters are directly measured to the same precision, the parameter set ensemble is spherical. The measurement precision required for well-constrained predictions is set by the stiffest direction. (C) If one parameter (here *θ*_2_) is known less precisely than the rest, the cloud is ellipsoidal. If not aligned with a sloppy direction, the cloud will admit many model behaviors and yield large prediction uncertainties. (Note that the aspect ratio of the real contours can be greater than 1,000.)

The difficulty of extracting precise parameter values from collective fits in systems biology modeling is well-known [26]. Sloppiness offers an explanation for this and predicts that it will be true even for fitting to complete data that the model can fit perfectly. In a collective fit, the parameter set ensemble samples from all sets of parameters for which the model behavior is consistent with the data. Because sloppy models are very insensitive to parameter combinations that lie along sloppy directions, the parameter set ensemble can extend very far in those directions, as illustrated schematically in Figure 2A. As a result, individual parameters can be very poorly determined (e.g., confidence interval indicated by Σ_1_ in Figure 2A). Below, we discuss a test of this prediction over all the models in our collection.

Unless one has direct interest in the kinetic constants for the underlying reactions, uncertainties in model predictions are generally more important than uncertainties in model parameters. The parameter set ensemble illustrated in Figure 2A yields large uncertainties on individual parameters, but can yield small uncertainties on predictions. While the fitting process allows the ensemble to expand along sloppy directions, the fit naturally constrains the ensemble along stiff directions, so that model behavior varies little within the ensemble, and predictions can be consequently tight.

Direct parameter measurements, on the other hand, will have uncertainties that are uncorrelated with the model's underlying stiff and sloppy directions. For example, if all parameter measurements are of the same precision, the parameter set ensemble is spherical, as illustrated in Figure 2B. For tight predictions, this ensemble must not cross many contours, so the required precision is set by the stiffest direction of the model. Consequently, high precision parameter measurements are required to yield tight predictions. Moreover, these measurements must be complete. If one parameter is known less precisely, the parameter set ensemble expands along that parameter axis, as illustrated in Figure 2C. If that axis is not aligned with a sloppy direction, model behavior will vary dramatically across the parameter set ensemble and predictions will have correspondingly large uncertainties. Below we discuss tests of both these notions, exploring the effects of direct parameter measurement uncertainty on predictions of a particular model.

### Parameter Values from Collective Fits

Does the sloppiness of these models really prevent one from extracting parameters from collective fits? Here we discuss a test of this prediction using an idealized fitting procedure.

Our *χ* ^2^ measure of model behavior change (Equation 1) corresponds to the cost function for fitting model parameters to continuous time-series data that the model fits perfectly at parameters *θ* ^*^; *H* ^χ^2^^ is the corresponding Fisher information matrix (Equation 2). We used this idealized situation to test the prediction that collective fits will often poorly constrain individual parameters in our collection of sloppy models.

**Figure 3:**
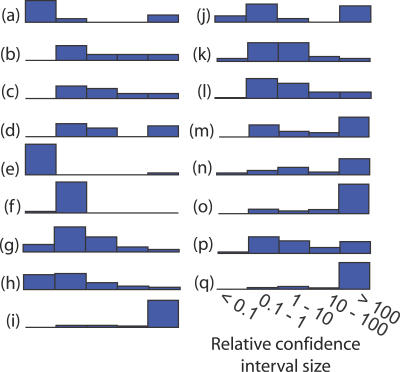
Fitting Parameters to Idealized Data Shown are histograms of the relative confidence interval size Σ for each parameter in each model of our collection, after fitting 100 times as many time-series data points (each with 10% uncertainty) as parameters. In most cases, a large number of parameters are left with greater than 100% uncertainty. (A parameter constrained with 95% probability to lie between 1 and 100 would have Σ ≈ 100.) Labels are as in Figure 1.

We defined the relative 95% confidence interval size Σ*_i_* as the ratio between parameter *i* at the upper and lower extremes of the interval, minus 1. (A parameter value constrained after the fit to lie between 10 and 1,000 would have Σ ≈ 100, while one constrained between 1.0 and 1.5 would have Σ = 0.5.) We assumed 100 times as many data points (each with 10% uncertainty) as the number of parameters in each model. Figure 3 shows histograms of the quadratic approximation to Σ for each parameter in each model after fitting such data (see Methods). For most of the models, Figure 3 indicates that such fitting leaves many parameters with greater than 100% uncertainty (Σ > 1). Indeed, even fitting this large amount of ideal data can leave many parameter values very poorly determined, as expected from the sloppiness of these models and our discussion of Figure 2A.

The fact that nonlinear multiparameter models often allow a wide range of correlated parameters to fit data has long been appreciated. As one example, a 1987 paper by Brodersen et al. on ligand binding to hemoglobin and albumin empirically found many sets of parameters that acceptably fit experimental data, with individual parameter values spanning huge ranges [13]. Our sloppy model perspective ([20,21,23], Figure 1) shows that there is a deep underlying universal pattern in such least-squares fitting. Indeed, an analysis of the acceptable binding parameter sets from the 1987 study shows the same characteristic sloppy eigenvalue spectrum we observed in Figure 1B (Text S5).

### Predictions from Direct Parameter Measurements

Figure 2B and 2C suggest that direct parameter measurements must be both precise and complete to usefully constrain predictions in sloppy systems. Here we discuss a test of this notion in a specific model.

**Figure 4:**
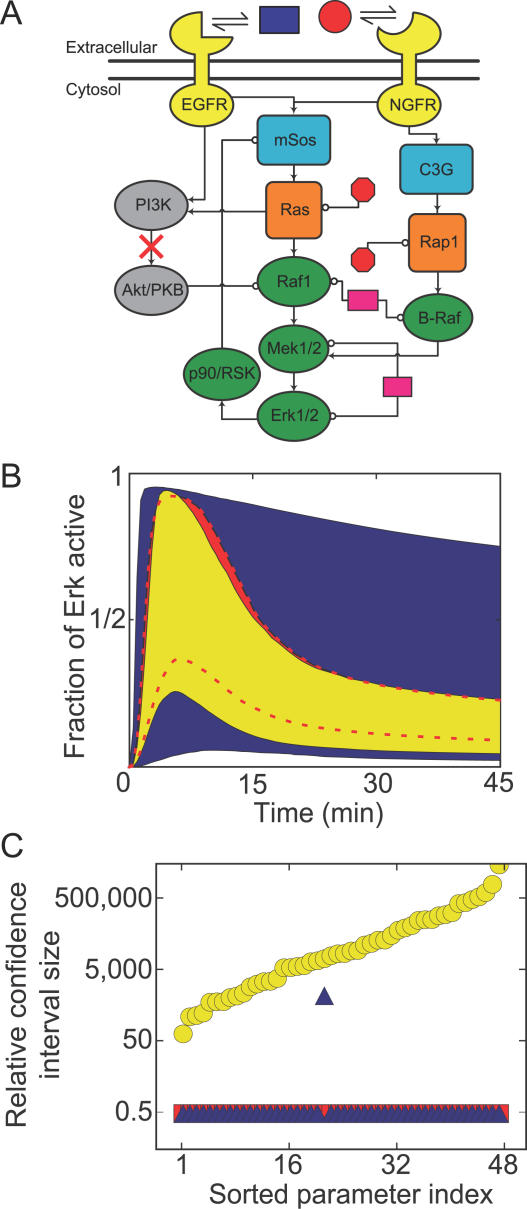
Parameter and Prediction Uncertainties (A) Our example prediction is for ERK activity upon EGF stimulation given PI3K inhibition in this 48-parameter model of growth-factor signaling in PC12 cells [21]. (B) Shaded regions are 95% confidence intervals calculated via exhaustive Monte Carlo for our example prediction given various scenarios for constraining parameter values. (C) Plotted is the relative size Σ of the 95% confidence interval for each parameter. The scenarios represented are: (red, squares) all model parameters individually measured to high precision, (blue, triangles) all parameters precisely measured, except one estimated to low precision, (yellow, circles) all parameters collectively fit to 14 real cell-biology experiments. Precisely measured individual parameter values enable a tight prediction, (B) middle red band; but even one poorly known parameter can destroy predictive power, (B) wide blue band. In contrast, the collective fit yields a tight prediction, (B) tightest yellow band; but only very loose parameter constraints, (C) circles. The large parameter uncertainties from the collective fit, (C) circles, calculated here by Monte Carlo are qualitatively similar to those seen in the linearized fit to idealized data (Figure 3, model (i)). (For clarity, the dashed red lines trace the boundary of the red confidence interval.)

We worked with the 48-parameter growth-factor-signaling model of Brown et al., shown schematically in Figure 4A [21]. The parameters in this model were originally collectively fit to 14 time-series cell-biology experiments. We focused on this model because it is instructive to compare our results concerning direct parameter measurements with prior results from collective fitting. For our analysis, we assumed that hypothetical direct parameter measurements would be centered about the original best-fit values.

One important test of the model was a prediction of the time-course of ERK activity upon EGF stimulation, given inhibition of the PI3K branch of the pathway. The yellow shaded region in Figure 4B shows the uncertainty bound on this prediction from the original collective fit, calculated by exhaustive Monte Carlo [21]. The tightness of this prediction is remarkable considering the huge uncertainties the collective fit left in the individual parameter values (yellow circles in Figure 4C). Not a single parameter was constrained to better than a factor of 50.

How precise would direct parameter measurements have to be to yield as tight a prediction as the collective fit? For this prediction, the PI3K branch (inhibited) and C3G branch (NGF-dependent) of the pathway are irrelevant in the model; the remaining reactions involve 24 parameters. To achieve the red prediction in Figure 4B, all 24 involved parameters must be measured to within a factor of plus or minus 25% (Figure 4C, red squares). With current techniques, measuring even a single in vivo biochemical parameter to such precision would be a challenging experiment. Such high precision is required because, as illustrated in Figure 2B, the measurements need to constrain the stiffest combination of model parameters.

What if a single parameter is left unmeasured? For example, consider high-precision measurements of 23 of the 24 involved parameters, all but the rate constant for the activation of Mek by Raf1. For this unmeasured parameter, we assumed that an informed estimate could bound it at 95% confidence to within a total range of 1,000 (e.g., between 1 *s* ^−1^ and 1,000 *s* ^−1^). The resulting prediction (blue in Figure 4B) has very large uncertainty and would likely be useless. Note that these hypothetical measurements constrain every individual parameter value more tightly than the original collective fit (blue triangles versus yellow circles in Figure 4C), yet the prediction is much less well-constrained. Neither this parameter nor this prediction is unique. Uncertainty for this prediction is large if any one of about 18 of the 24 involved parameters is unmeasured (Text S2). Furthermore, other possible predictions in this model are similarly fragile to single unmeasured parameters (Text S3).

To usefully constrain Brown et al.'s model, direct parameter measurements would need to be both precise and complete. By contrast, collective parameter fitting yielded tight predictions with only a modest number of experiments. These results are expected given the model's sloppiness.

## Discussion

By examining 17 models from the systems biology literature [2,21,25,28–41], we showed that their parameter sensitivities all share striking common features deemed “sloppiness”; the sensitivity eigenvalues span many decades roughly evenly (Figure 1B) and tend not to be aligned with single parameters (Figure 1C). We argued that sloppy parameter sensitivities help explain the difficulty of extracting precise parameter estimates from collective fits, even from comprehensive data. Additionally, we argued that direct parameter measurements should be inefficient at constraining predictions from sloppy models. We then showed that collective parameter fits to complete time-series data do indeed yield large parameter uncertainties in our model collection (Figure 3). Finally, we confirmed for the 48-parameter signaling model of Brown et al. [21] that direct parameter measurements must be formidably precise and complete to usefully constrain model predictions (Figure 4).

What causes sloppiness? (1) Fundamentally, sloppiness involves an extraordinarily singular coordinate transformation in parameter space between the bare parameters natural in biology (e.g., binding affinities and rate constants) and the eigenparameters controlling system behavior, as discussed in [23]. Both experimental interventions and biological evolution work in the bare parameter space, so this parameterization is fundamental to the system, not an artifact of the modeling process. (2) Sloppiness depends not just upon the model, but also on the data it is fit to; exhaustive experiments designed to decouple the system and separately measure each parameter will naturally not yield sloppy parameter sensitivities. (3) In biological systems fit to time-series data, Brown and Sethna [20] note that sloppiness may arise due to underdetermined systems, proximity to bifurcations, and separation of time or concentration scales, but they doubt that these can explain all the sloppiness found in their model. Our analysis includes complete data on all species, and hence is overdetermined. Small eigenvalues near bifurcations are associated with dynamic variables, and not the system parameters we investigate. To study the effect of time and concentration scales, we calculated *H* ^χ^2^^ for a version of the Brown et al. model in which all concentrations and rate constants were scaled to 1. The resulting model remains sloppy, with eigenvalues roughly uniformly spanning five decades (Text S4). (4) Motivated by simple example systems, we have argued elsewhere that sloppiness emerges from a redundancy between the effects of different parameter combinations [23]. We are presently investigating decompositions of parameter space into sloppy subsystems [46] and the use of physically or biologically motivated nonlinear coordinate changes to remove sloppiness or motivate simpler models. These potential methods for model refinement, however, demand a complete and sophisticated understanding of the system that is unavailable for many biological systems of current interest.

Parameter estimation has been a serious obstacle in systems biology modeling. With tens of unknown parameters, a typical modeling effort might draw some values from the literature (possibly from in vitro measurements or different cell lines) [33,38], set classes of constants to the same value (e.g., phosphorylation rates) [31,32,41], and adjust key parameters to qualitatively best fit the existing data [2,37,40]. In retrospect, these approaches may be successful because the models are sloppy—they can be tuned to reality by adjusting one key parameter per stiff direction, independently of how reliably the other parameters are estimated.

Computational modeling is a potentially invaluable tool for extrapolating from current experiments and distinguishing between models. But we cannot trust the predictions of these models without testing how much they depend on uncertainties in these estimated parameters. Conversely, if we insist upon a careful uncertainty analysis, it would seem unnecessary to insist upon tight prior estimates of the parameters, since they do not significantly enhance model predictivity. Because the behavior of a sloppy model is dominated by a few stiff directions that nonetheless involve almost all the parameters, direct parameter measurements constrain predictions much less efficiently than comparably difficult experiments probing collective system behavior.

Our suggestion of making predictions from models with very poorly known parameters may appear dangerous. A model with tens or hundreds of unmeasured parameters might seem completely untrustworthy; we certainly believe that any prediction derived solely from a best-fit set of parameters is of little value. Uncertainty bounds derived from rigorous sensitivity analysis, however, distinguish those predictions that can be trusted from those that cannot. Of course, successful fits and predictions may arise from models that are incorrect in significant ways; for example, one model pathway with adjusted parameters may account for two parallel pathways in the real system. A model that is wrong in some details may nevertheless be useful in guiding and interpreting experiments. For computational modeling to be useful in incompletely understood systems, we must focus not on building the final, perfect, model with all parameters precisely determined, but on building incomplete, tentative, and falsifiable models in the most expressive and predictive fashion feasible.

Given that direct parameters measurements do not efficiently constrain model behavior, how do we suggest that experimentalists decide what experiment to do next? If the goal is to test the assumptions underlying a model, one should look for predictions with tight uncertainty estimates that can be readily tested experimentally. If the goal is to reduce uncertainty in crucial model predictions, one must invoke the statistical methods of optimal experimental design, which we have studied elsewhere [27] and which may be conveniently implemented in modeling environments that incorporate sensitivity analysis (such as SloppyCell [44]).

In our approach, the model and its parameters cannot be treated in isolation from the data that informed model development and parameter fitting. This complicates the task of exchanging knowledge in the modeling community. To support our approach, standards such as SBML [43] that facilitate automated model exchange will need to be extended to facilitate automated data exchange.

Every one of the 17 systems biology models we studied exhibits a sloppy spectrum of parameter sensitivity eigenvalues; they all span many decades roughly evenly and tend not be aligned with single parameters. This striking and apparently universal feature has important consequences for the modeling process. It suggests that modelers would be wise to try collective parameter fits and to focus not on the quality of their parameter values but on the quality of their predictions.

## Methods

### Hessian computations.

*H* ^χ^2^^ can be calculated as

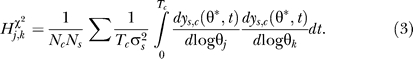



Second derivative terms (*d* ^2^ *y_s_*_,*c*_(*θ* ^*^,*t*) / *d*log*θ_i_ d*log*θ_j_*) might be expected, but they vanish because we evaluate *H* ^χ^2^^ at *θ* ^*^. Equation 3 is convenient because the first derivatives (*dy_s_*_,*c*_(*θ* ^*^,*t*) / *d* log *θ_j_*) can be calculated by integrating sensitivity equations. This avoids the use of finite-difference derivatives, which are troublesome in sloppy systems.

The projections *P_i_* of the ellipsoids shown in Figure 2A onto bare parameter axis *i* are proportional to 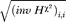 . The intersections *I_i_* with axis *i* are proportional to 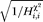 , with the same proportionality constant.

### Parameter uncertainties.

To rescale *H* ^χ^2^^ so that it corresponds to fitting *N_d_* data points, each with uncertainty a fraction *f* of the species' maximal value, we multiply *H* ^χ^2^^ by *N_d_* / *f* ^2^. In the quadratic approximation, the one-standard-deviation uncertainty in the logarithm of parameter *θ_i_* after such a collective fit is given by 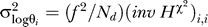 . The relative size of the 95% confidence interval is then 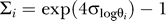 .

### Prediction uncertainties.

The red and blue prediction uncertainties shown in Figure 4B were calculated by randomly generating 1,000 parameter sets consistent with the stated parameter uncertainties. (For each parameter *i*, the logarithm of its value is drawn from a normal distribution with mean 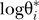 and standard deviation 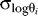 specified by the desired Σ.) For each parameter set, the Erk time course was calculated, and at each time-point the shaded regions in the figure contain the central 95% of the time courses.

### Software.

All computations were performed in the open-source modeling environment SloppyCell, version 0.81 [44]. SBML files and SloppyCell scripts to reproduce all presented calculations are in Dataset S1.

## Supporting Information

Dataset S1 SBML Files, SloppyCell Scripts, and *H* ^χ^2^^ Hessians (1.1 MB ZIP)

Text S1 Stiffest Eigenvectors (112 KB PDF)

Text S2 Effect of Other Poorly Determined Parameters (94 KB PDF)

Text S3 Fragility of Other Predictions (48 KB PDF)

Text S4 Rescaled Model of Brown et al. (46 KB PDF)

Text S5 Eigenvalue Analysis of Brodersen et al. Binding Studies (42 KB PDF)

### Accession Numbers

Models discussed that appear in the BioModels database [42] are: (a) BIOMD0000000005, (c) BIOMD0000000003, (d) BIOMD0000000035, (e) BIOMD0000000002, (f) BIOMD0000000010, (h) BIOMD0000000021, (i) BIOMD0000000033, (k) BIOMD0000000022, (l) BIOMD0000000055, (n) BIOMD0000000015, (o) BIOMD0000000051, (p) BIOMD0000000056, (q) BIOMD0000000049.

## Abbreviations

SBML: Systems Biology Markup Language
