# Supplementary material for: Universally Sloppy Parameter Sensitivities in Systems Biology Models
Source: PLoS Comput Biol. 2007 Oct 5;3(10):e189. doi: 10.1371/journal.pcbi.0030189 (PMC2000971; doi:10.1371/journal.pcbi.0030189)
Supplement: Supplementary file 1 [file pcbi.0030189.sd002.pdf]

Universally Sloppy Parameter Sensitivities in Systems Biology Models
Supporting Text 1: Stiffest Eigenvectors

Ryan N. Gutenkunst, Joshua J. Waterfall, Fergal P. Casey,
Kevin S. Brown, Christopher R. Myers, and James P. Sethna

The following figures show the four stiffest eigenvectors of H^{χ^2} (corresponding to the four largest eigenvalues) for each model in our collection. In each eigenvector the five parameters with the largest contributions are labeled. With few exceptions, the eigenvectors tend to be inscrutable combinations of many parameters.

---

[1] Tyson JJ (1991) Modeling the cell division cycle: cdc2 and cyclin interactions. Proc Natl Acad Sci USA 88:7328–7332.

[2] Zwolak JW, Tyson JJ, Watson LT (2005) Globally optimised parameters for a model of mitotic control in frog egg extracts. Syst Biol (Stevenage) 152:81–92.

[3] Goldbeter A (1991) A minimal cascade model for the mitotic oscillator involving cyclin and cdc2 kinase. Proc Natl Acad Sci USA 88:9107–9111.

[4] Vilar JMG, Kueh HY, Barkai N, Leibler S (2002) Mechanisms of noise-resistance in genetic oscillators. Proc Natl Acad Sci USA 99:5988–5992.

[5] Edelstein SJ, Schaad O, Henry E, Bertrand D, Changeux JP (1996) A kinetic mechanism for nicotinic acetylcholine receptors based on multiple allosteric transitions. Biol Cybern 75:361–379.

[6] Kholodenko BN (2000) Negative feedback and ultrasensitivity can bring about oscillations in the mitogen-activated protein kinase cascades. Eur J Biochem 267:1583–1588.

[7] Lee E, Salic A, Krüger R, Heinrich R, Kirschner MW (2003) The roles of APC and Axin derived from experimental and theoretical analysis of the Wnt pathway. PLoS Biol 1:e10.

[8] Leloup JC, Goldbeter A (1999) Chaos and birhythmicity in a model for circadian oscillations of the PER and TIM proteins in drosophila. J Theor Biol 198:445–459.

[9] Brown KS, Hill CC, Calero GA, Myers CR, Lee KH, et al. (2004) The statistical mechanics of complex signaling networks: nerve growth factor signaling. Phys Biol 1:184–195.

[10] von Dassow G, Meir E, Munro EM, Odell GM (2000) The segment polarity network is a robust developmental module. Nature 406:188–192.

[11] Ueda HR, Hagiwara M, Kitano H (2001) Robust oscillations within the interlocked feedback model of Drosophila circadian rhythm. J Theor Biol 210:401–406.

[12] Locke JCW, Southern MM, Kozma-Bognr L, Hibberd V, Brown PE, et al. (2005) Extension of a genetic network model by iterative experimentation and mathematical analysis. Mol Syst Biol 1:0013.

[13] Zak DE, Gonye GE, Schwaber JS, Doyle FJ III (2003) Importance of input perturbations and stochastic gene expression in the reverse engineering of genetic regulatory networks: insights from an identifiability analysis of an in silico network. Genome Res 13:2396–2405.

[14] Curto R, Voit EO, Sorribas A, Cascante M (1998) Mathematical models of purine metabolism in man. Math Biosci 151:1–49.

[15] Chassagnole C, Noisommit-Rizzi N, Schmid JW, Mauch K, Reuss M (2002) Dynamic modeling of the central carbon metabolism of *Escherichia coli*. Biotechnol Bioeng 79:53–73.

[16] Chen KC, Calzone L, Csikasz-Nagy A, Cross FR, Novak B, et al. (2004) Integrative analysis of cell cycle control in budding yeast. Mol Biol Cell 15:3841–3862.

[17] Sasagawa S, Ozaki Y, Fujita K, Kuroda S (2005) Prediction and validation of the distinct dynamics of transient and sustained ERK activation. Nat Cell Biol 7:365–373.

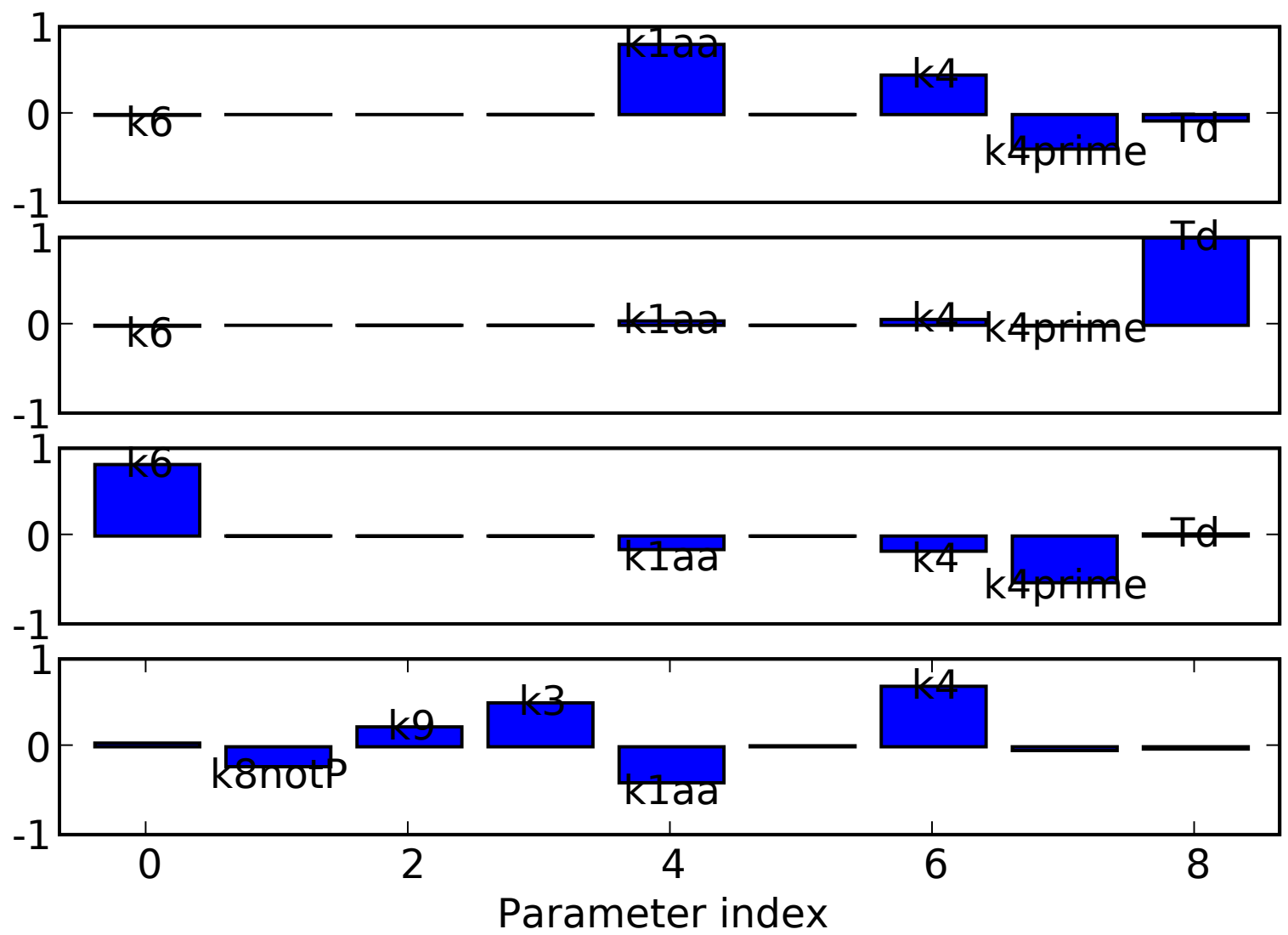

FIG. 1: Model (a): Tyson's model of the eukaryotic cell cycle [1].

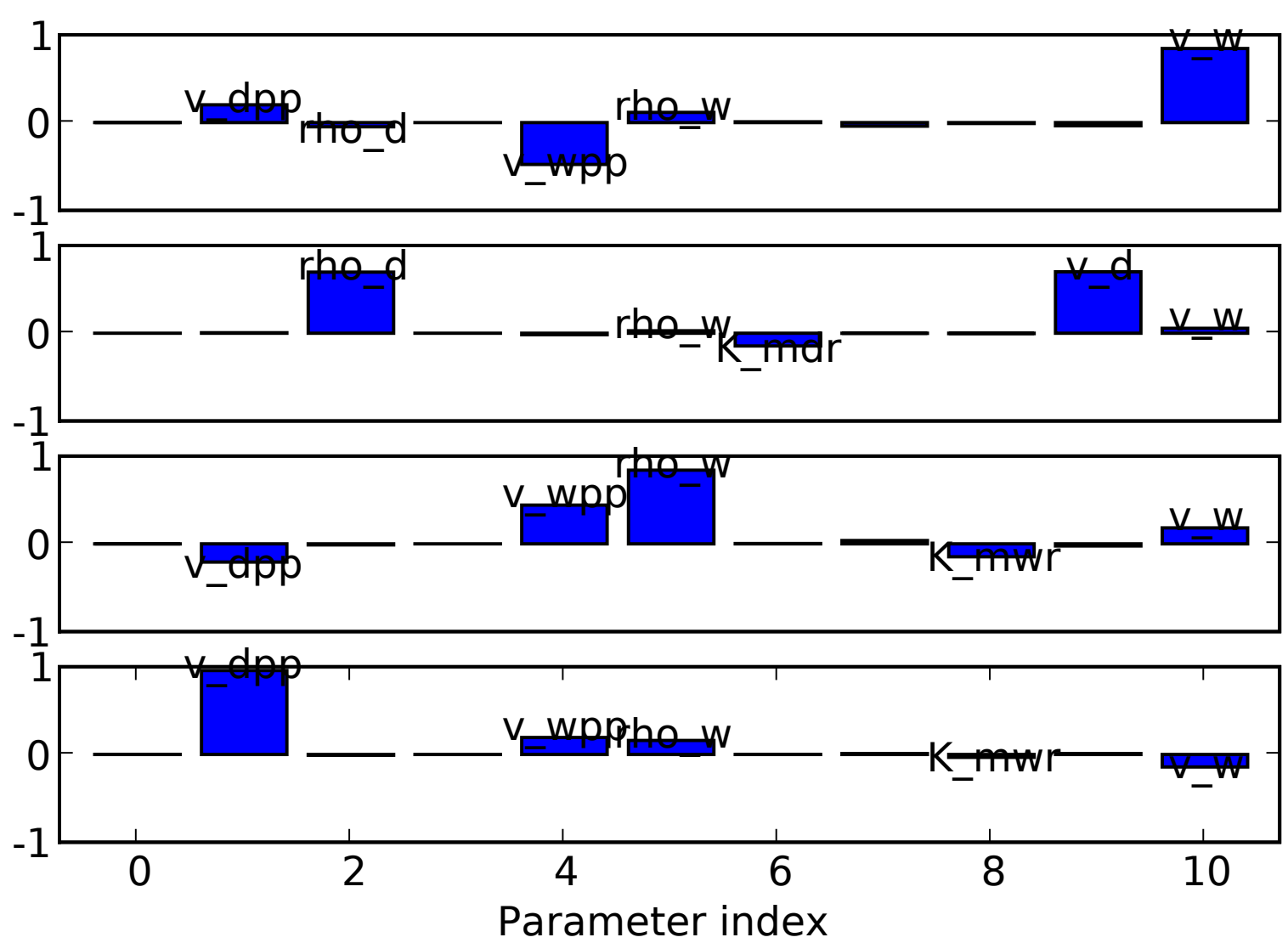

FIG. 2: Model (b): Zwolak *et al.*'s model of the Xenopus egg cell cycle [2].

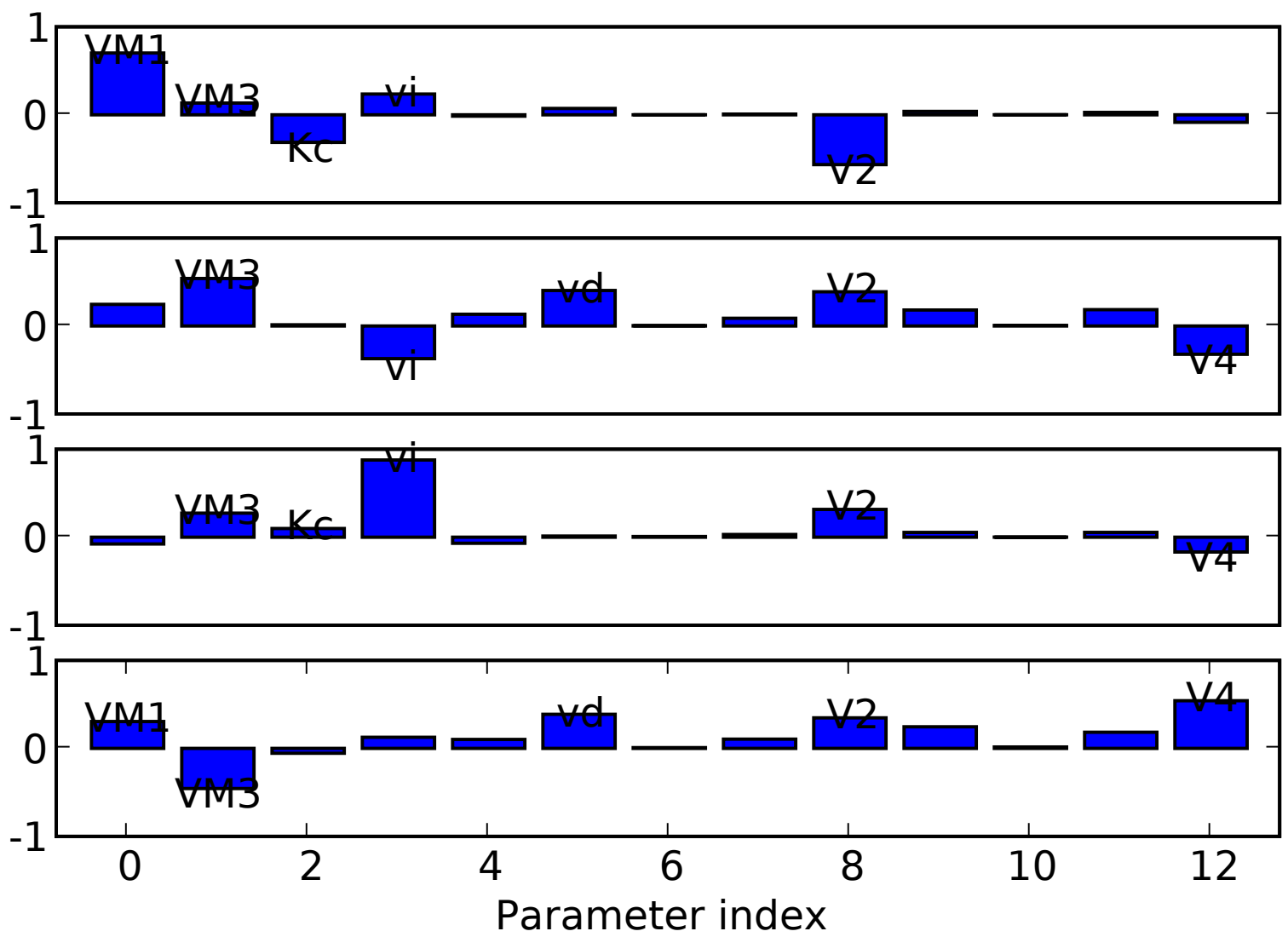

FIG. 3: Model (c): Goldbeters's model of eukaryotic mitosis [3].

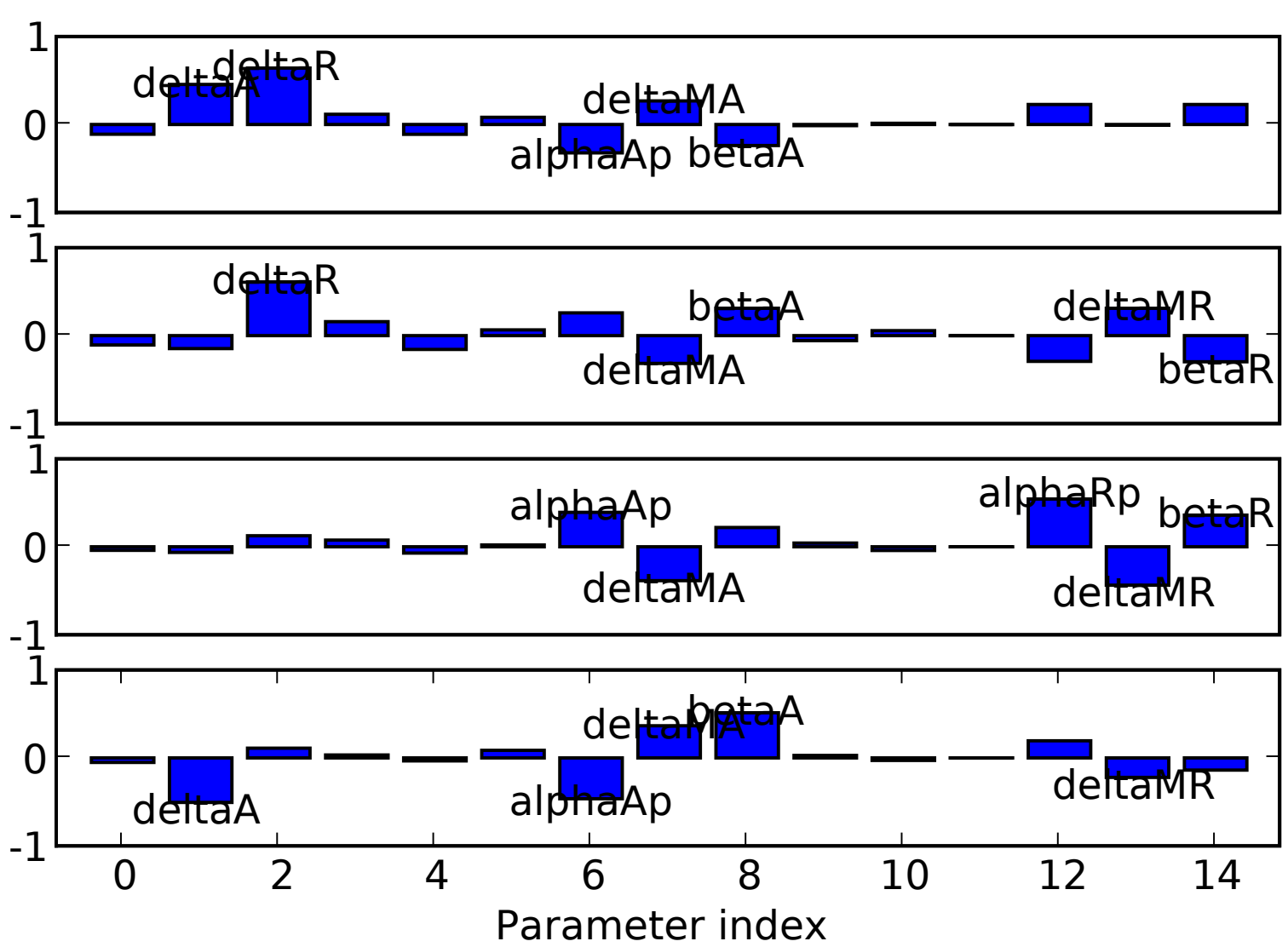

FIG. 4: Model (d): Vilar *et al.*'s generic circadian rhythm model [4].

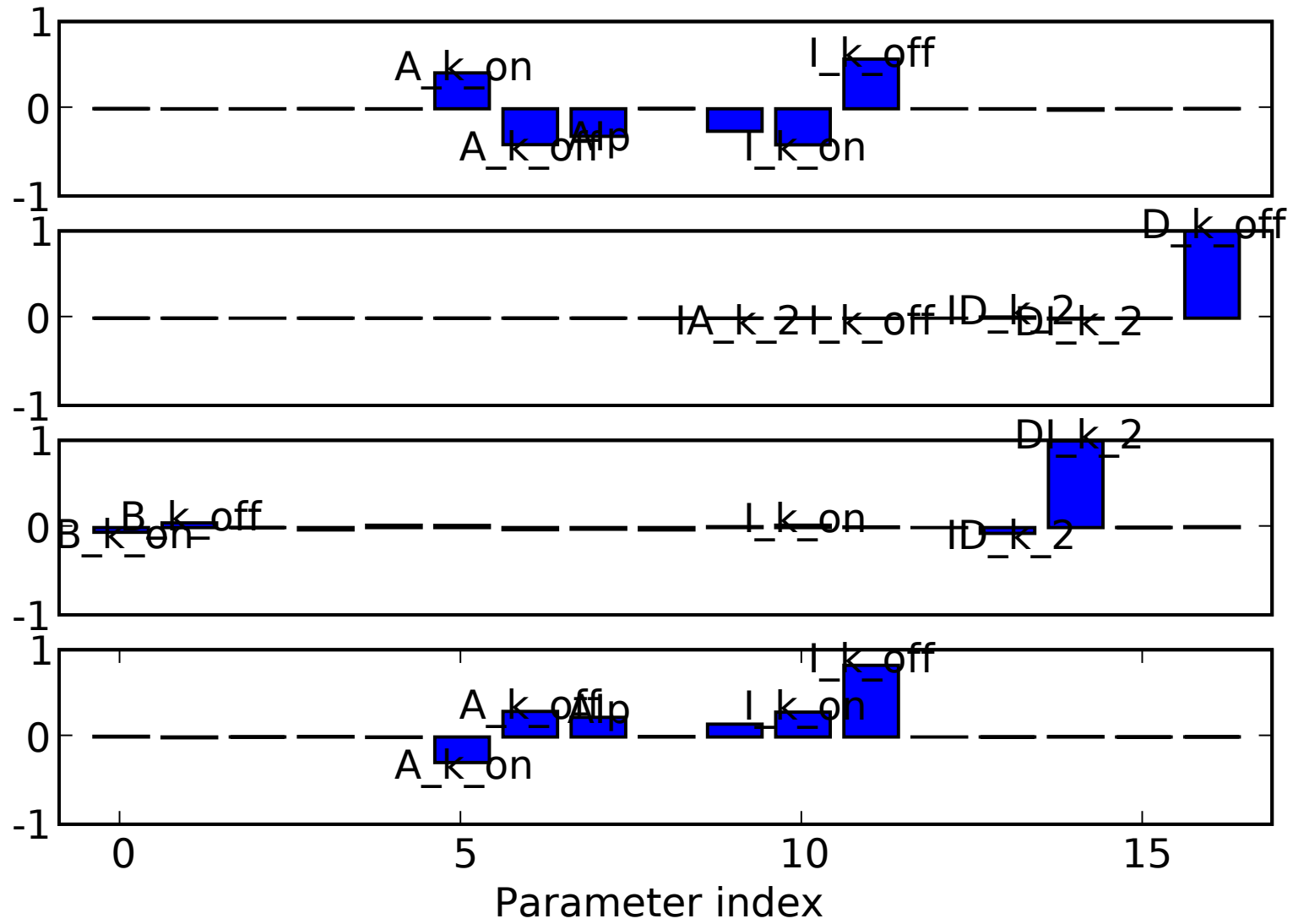

FIG. 5: Model (e): Edelstein *et al.*'s model of nicotinic acetylcholine intra-receptor dynamics [5].

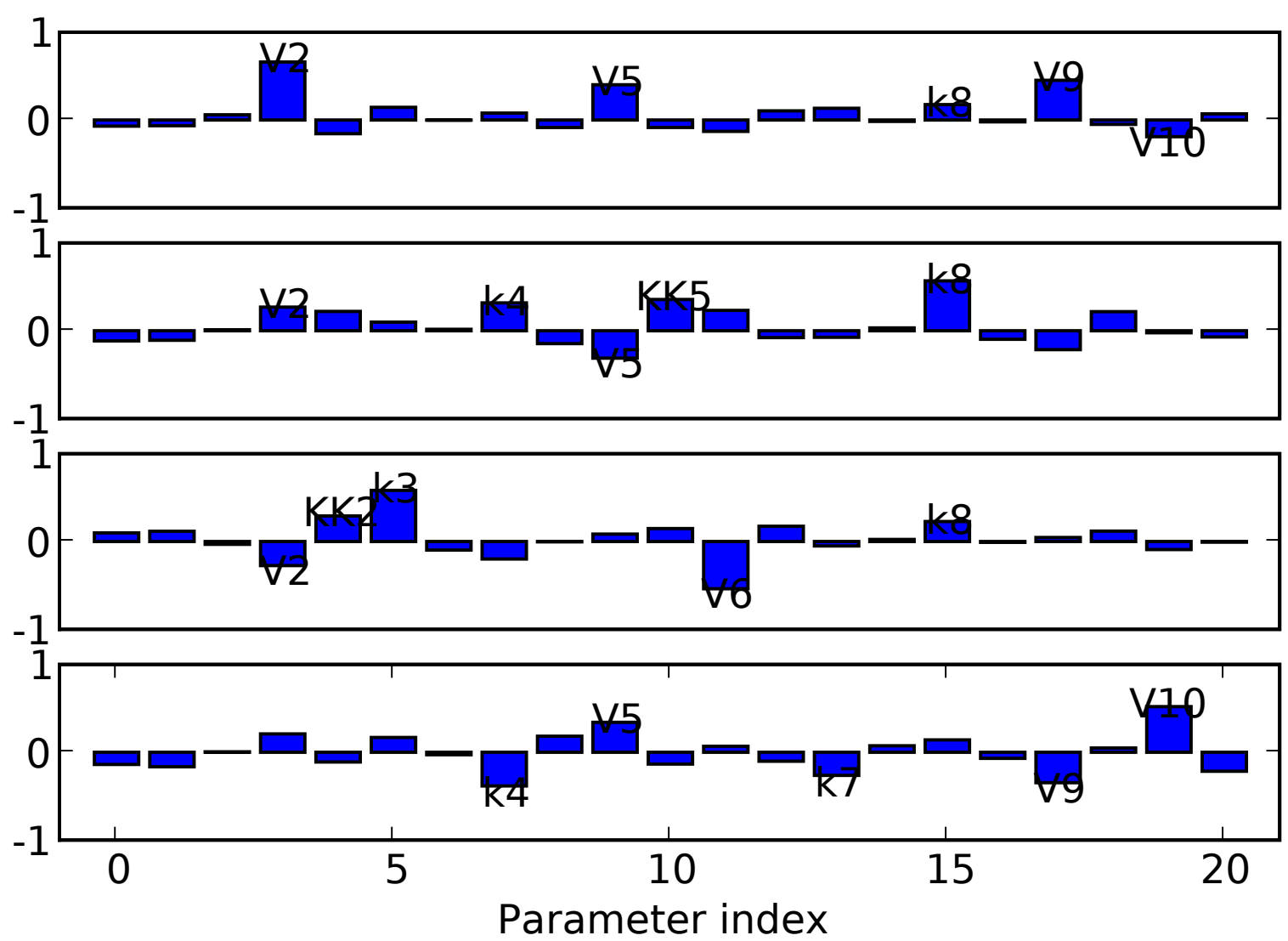

FIG. 6: Model (f): Kholodenko's model of a generic kinase cascade [6].

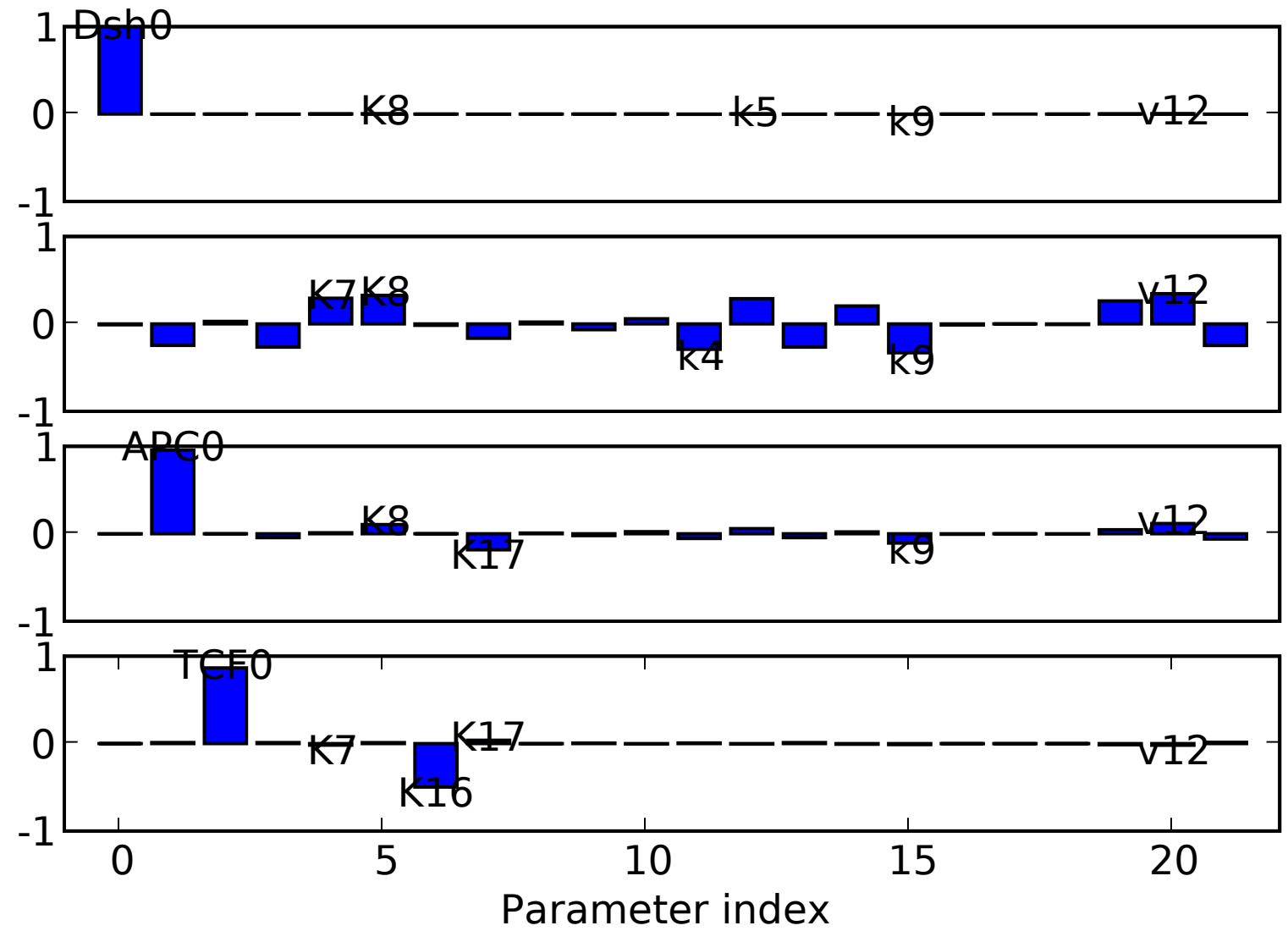

FIG. 7: Model (g): Lee *et al.*'s model of Xenopus Wnt signaling [7].

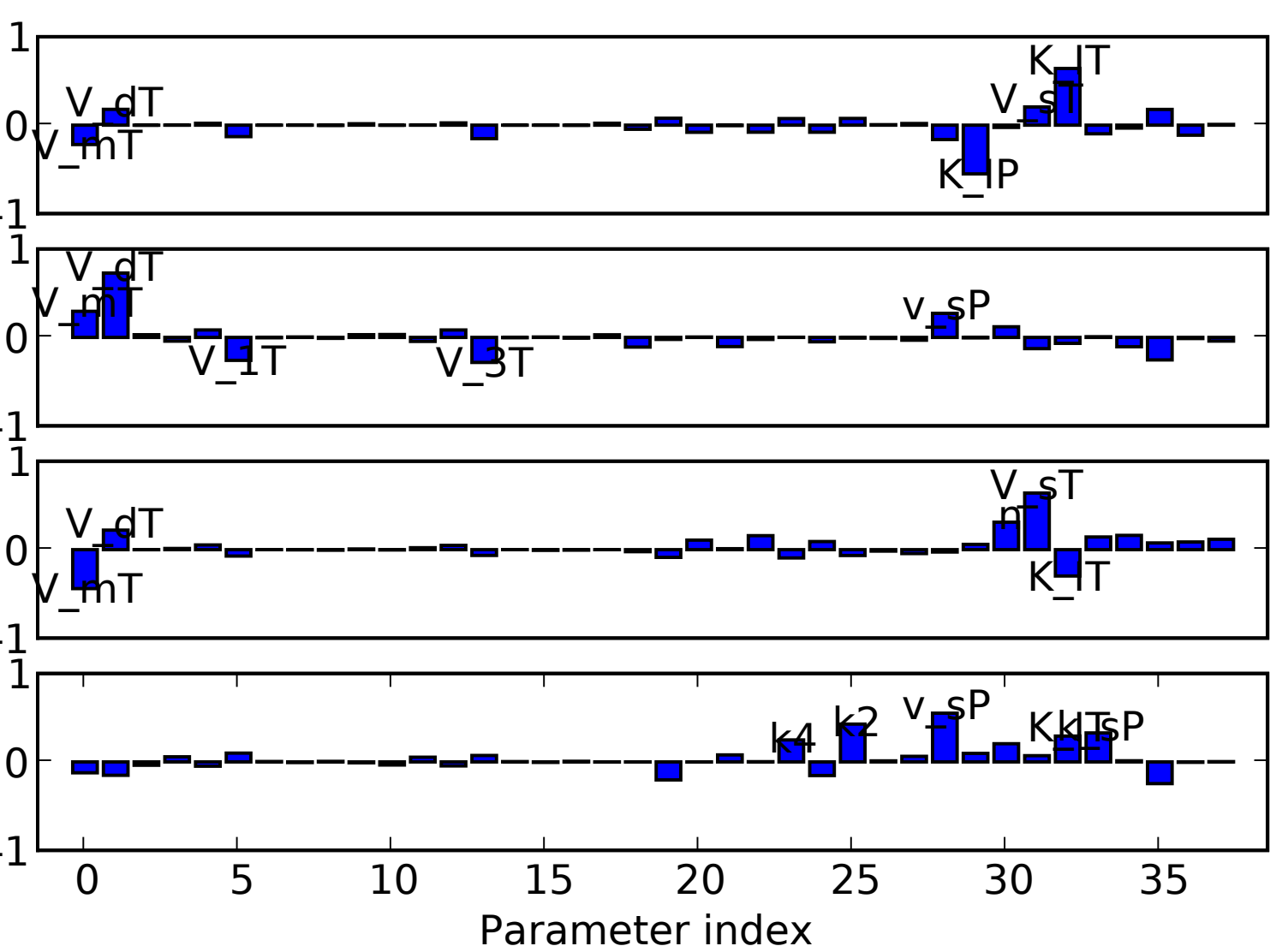

FIG. 8: Model (h): Leloup and Goldbeters's model of Drosophila circadian rhythm [8].

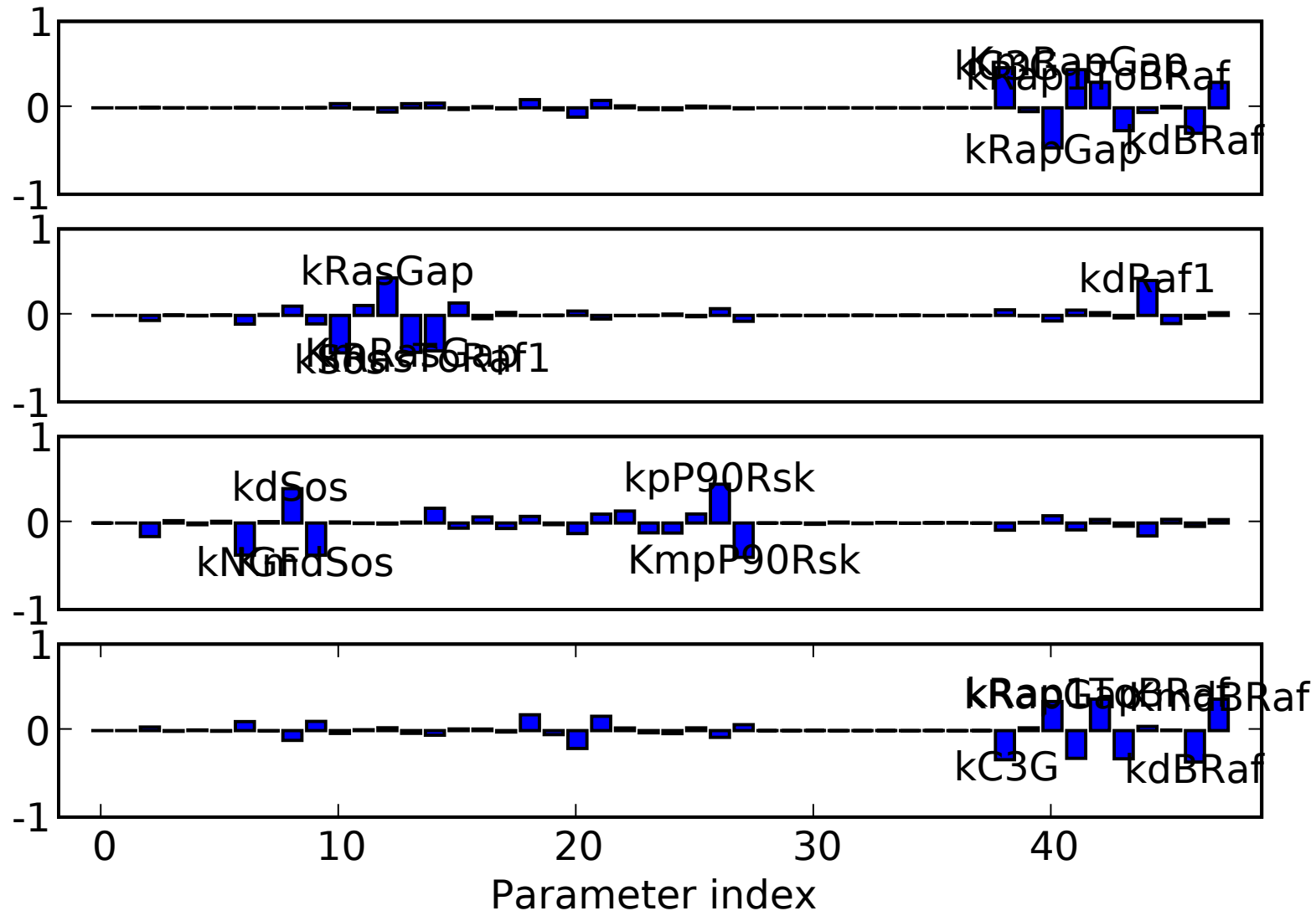

FIG. 9: Model (i): Brown *et al.*'s model of rat growth-factor signaling [9].

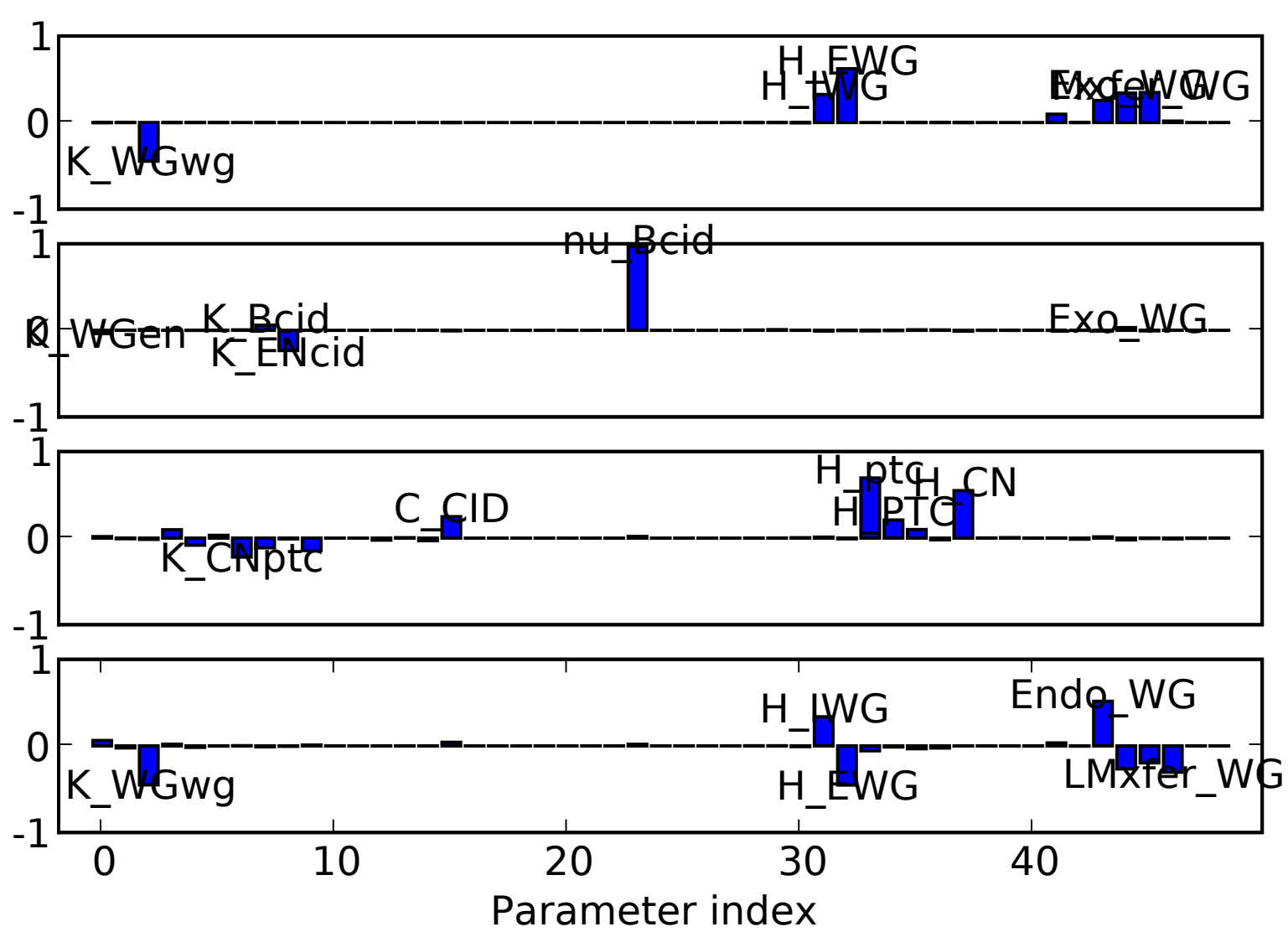

FIG. 10: Model (j): von Dassow *et al.*'s model of the Drosophila segment polarity network [10].

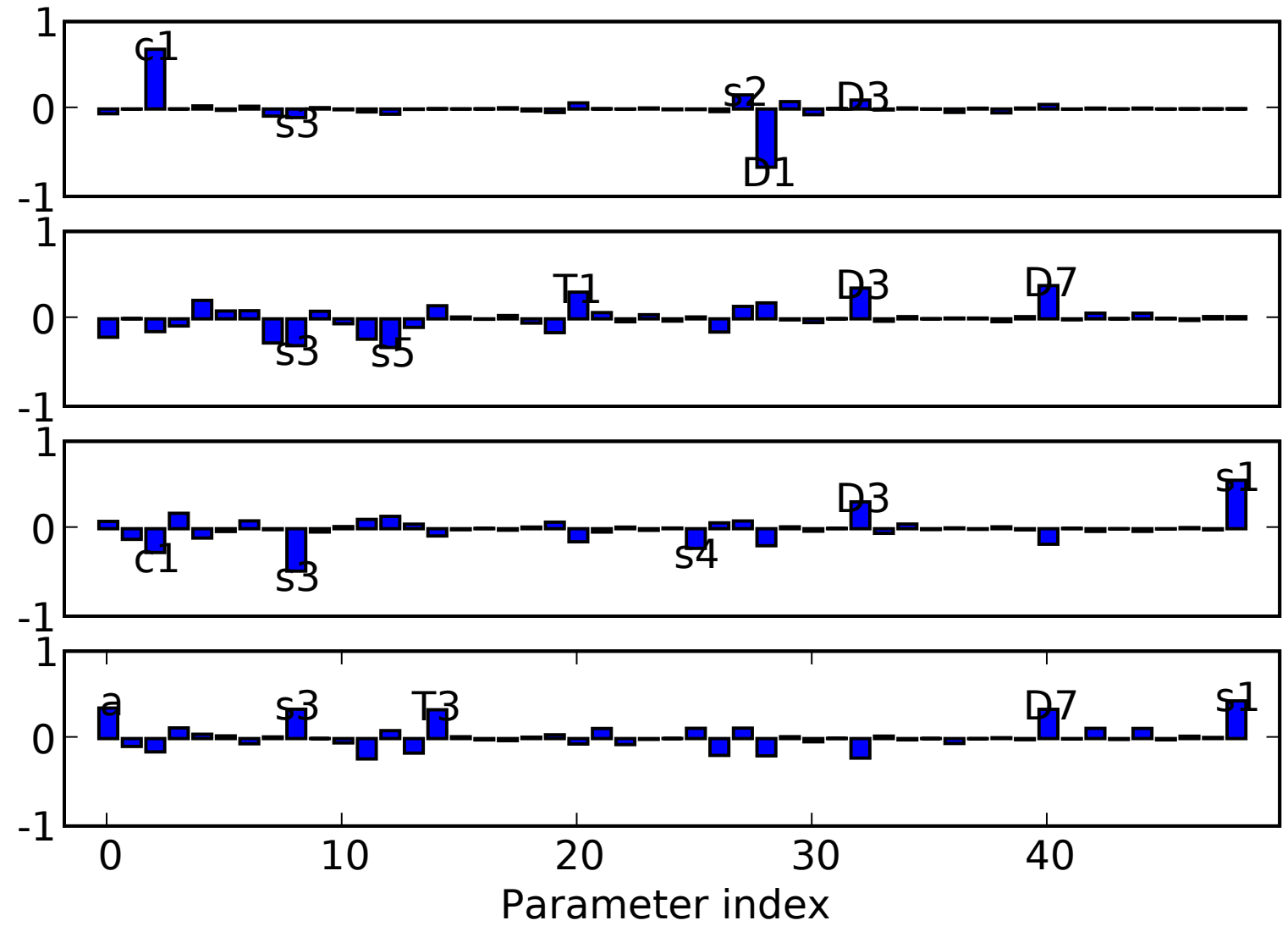

FIG. 11: Model (k): Ueda *et al.*'s model of Drosophila circadian rhythm [11].

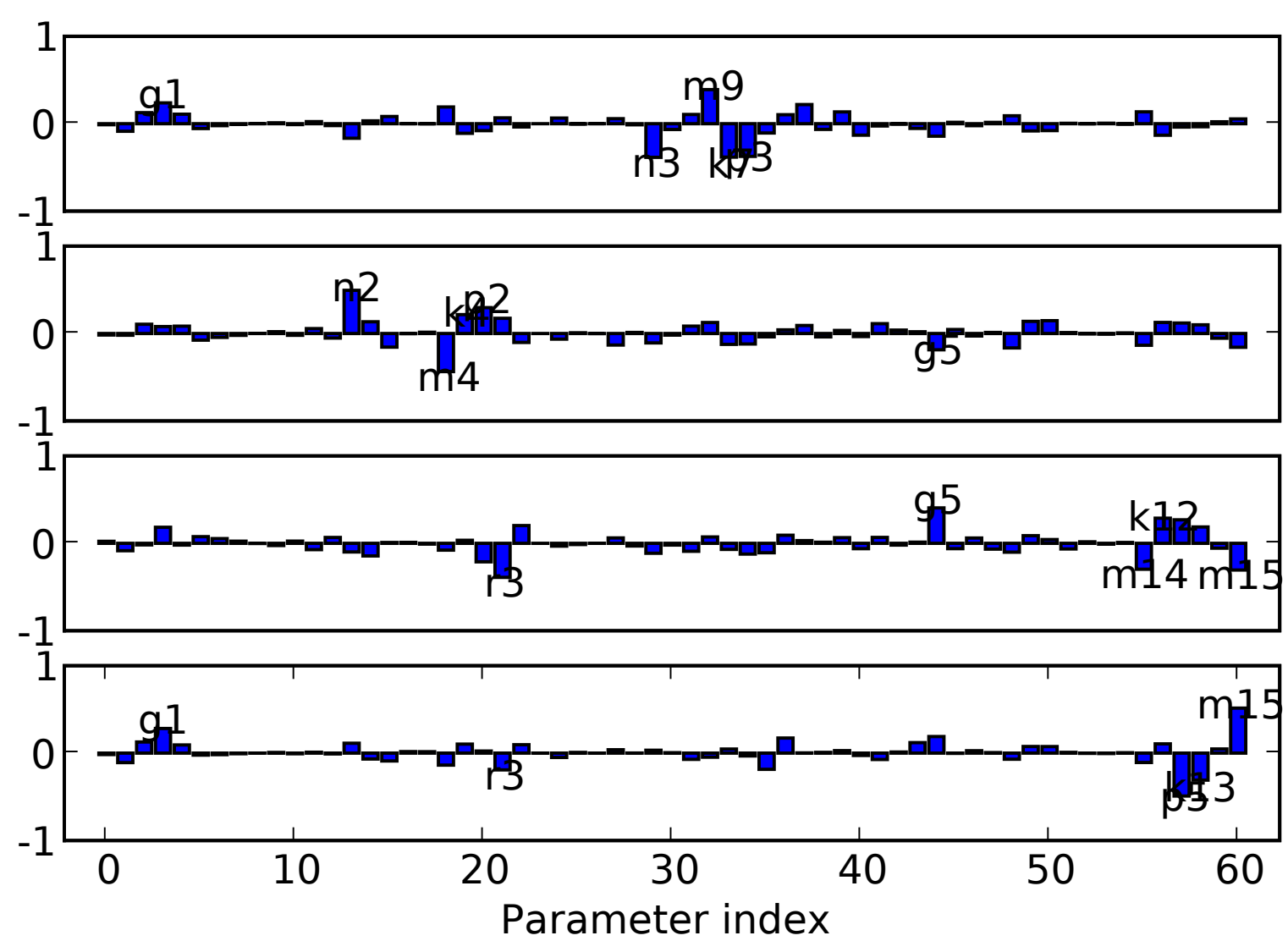

FIG. 12: Model (l): Locke *et al.*'s model of Arabidopsis circadian rhythm [12].

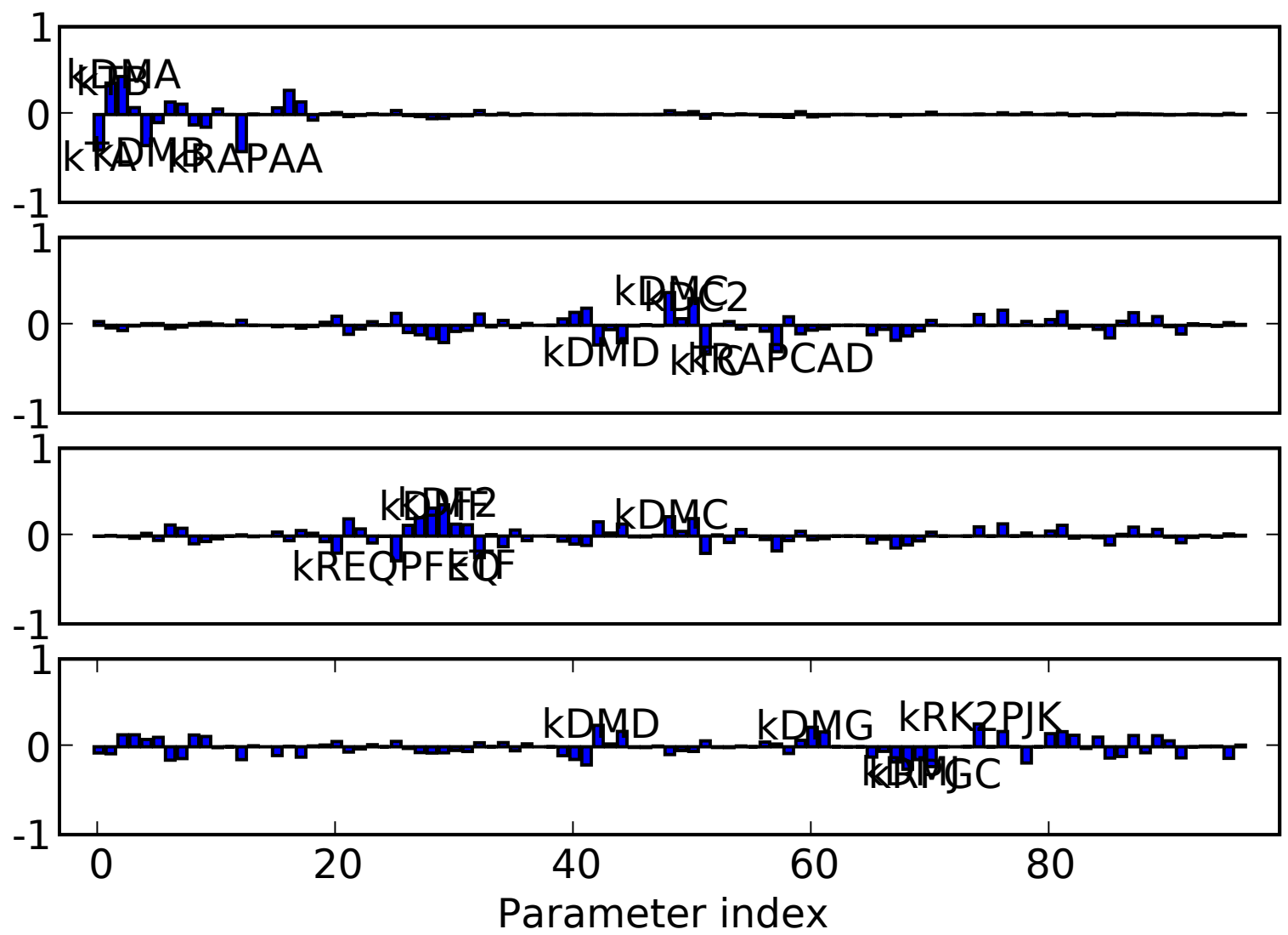

FIG. 13: Model (m): Zak *et al.*'s model of an *in silico* regulatory network [13].

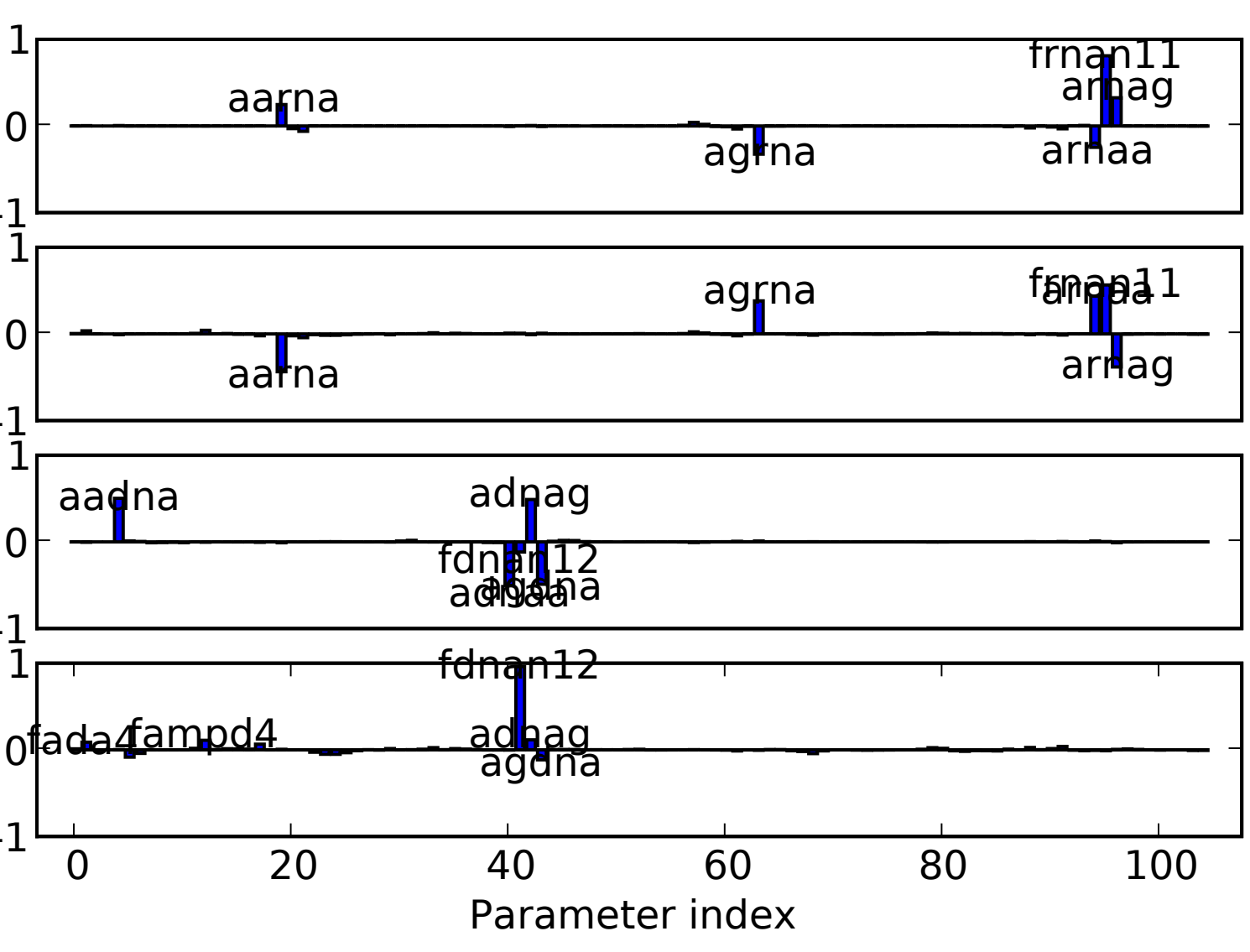

FIG. 14: Model (n): Curto *et al.*'s model of human purine metabolism [14].

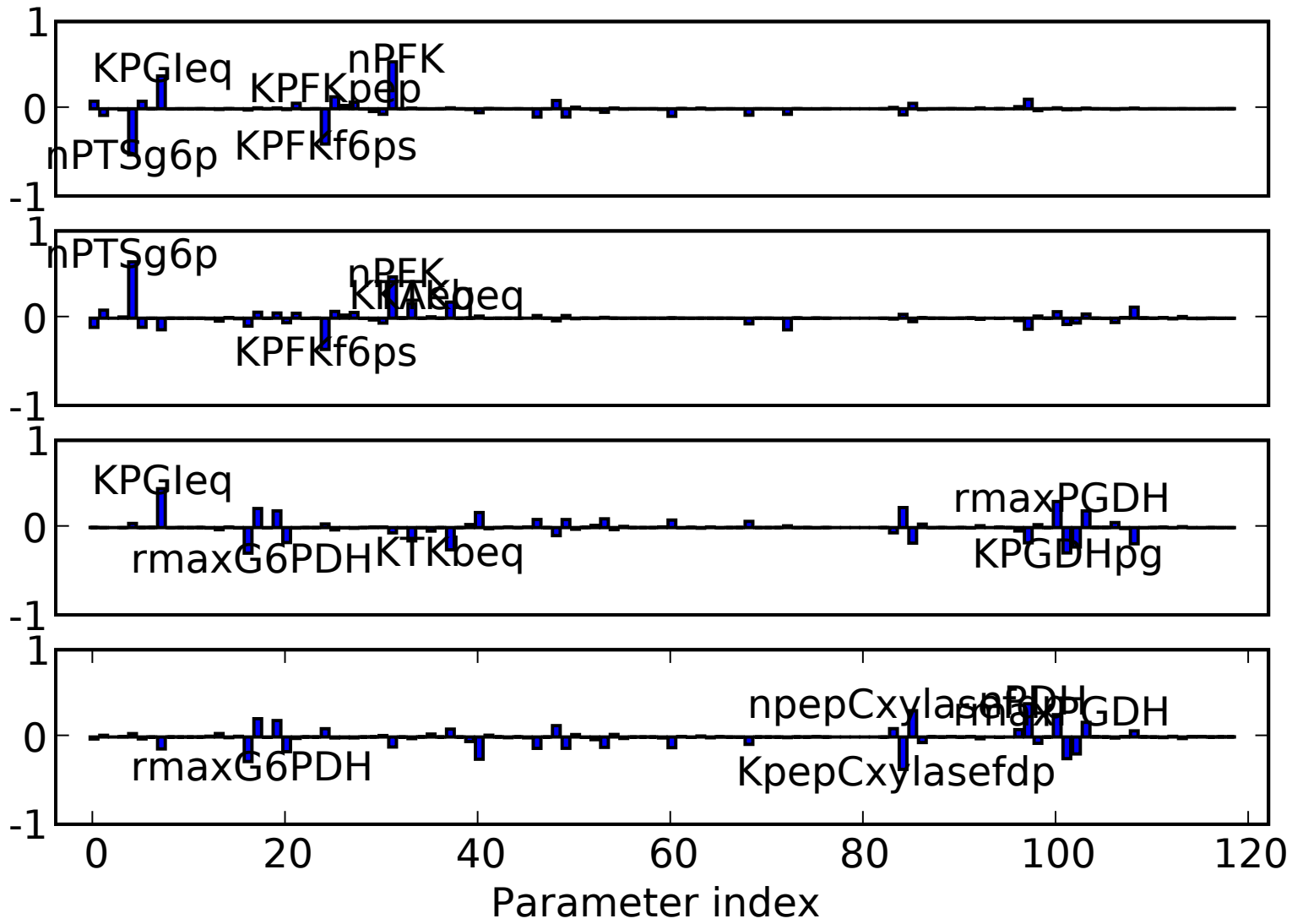

FIG. 15: Model (o): Chassagnole *et al.*'s model of E. coli carbon metabolism [15].

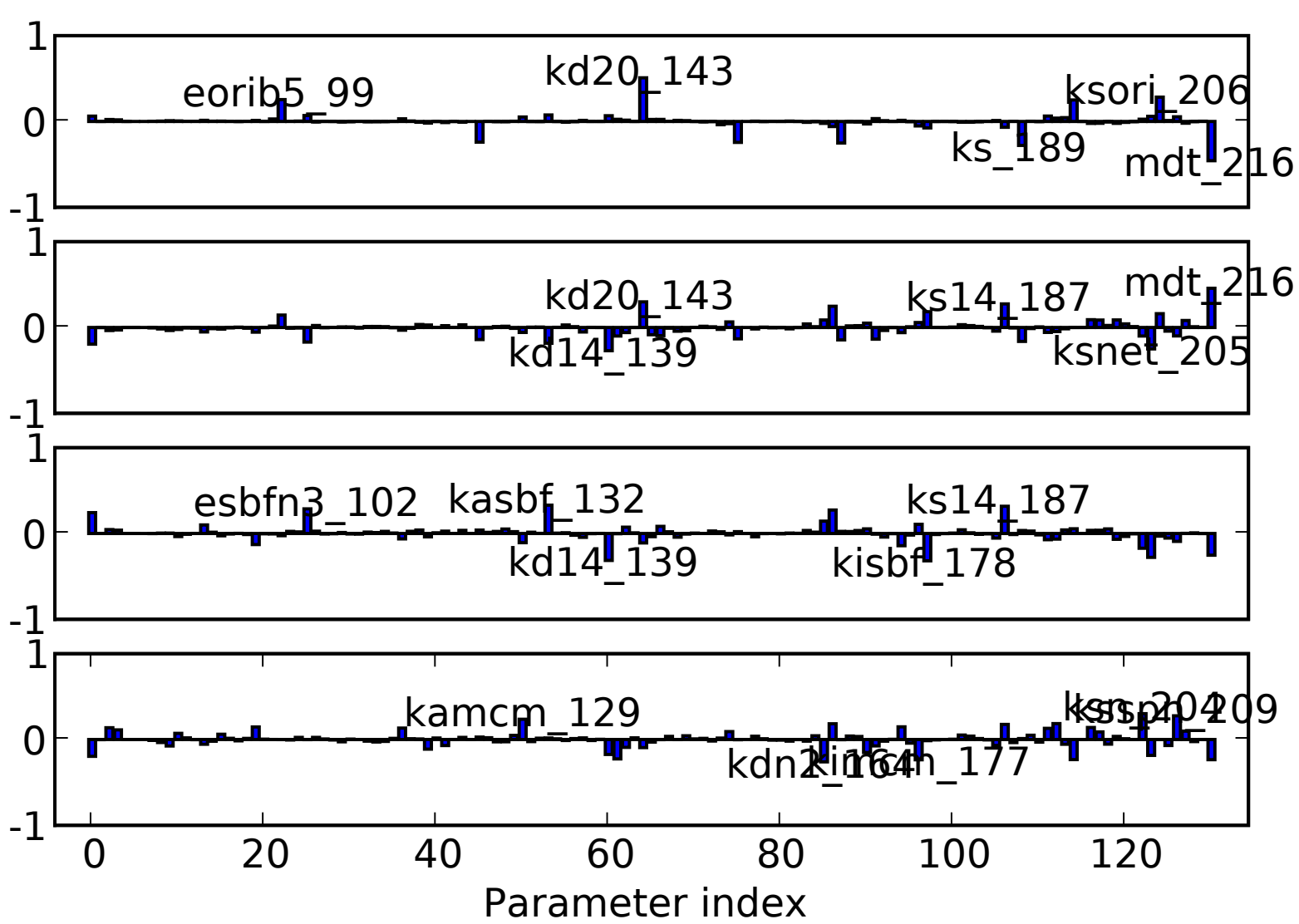

FIG. 16: Model (p): Chen *et al.*'s model of the budding yeast cell cycle [16].

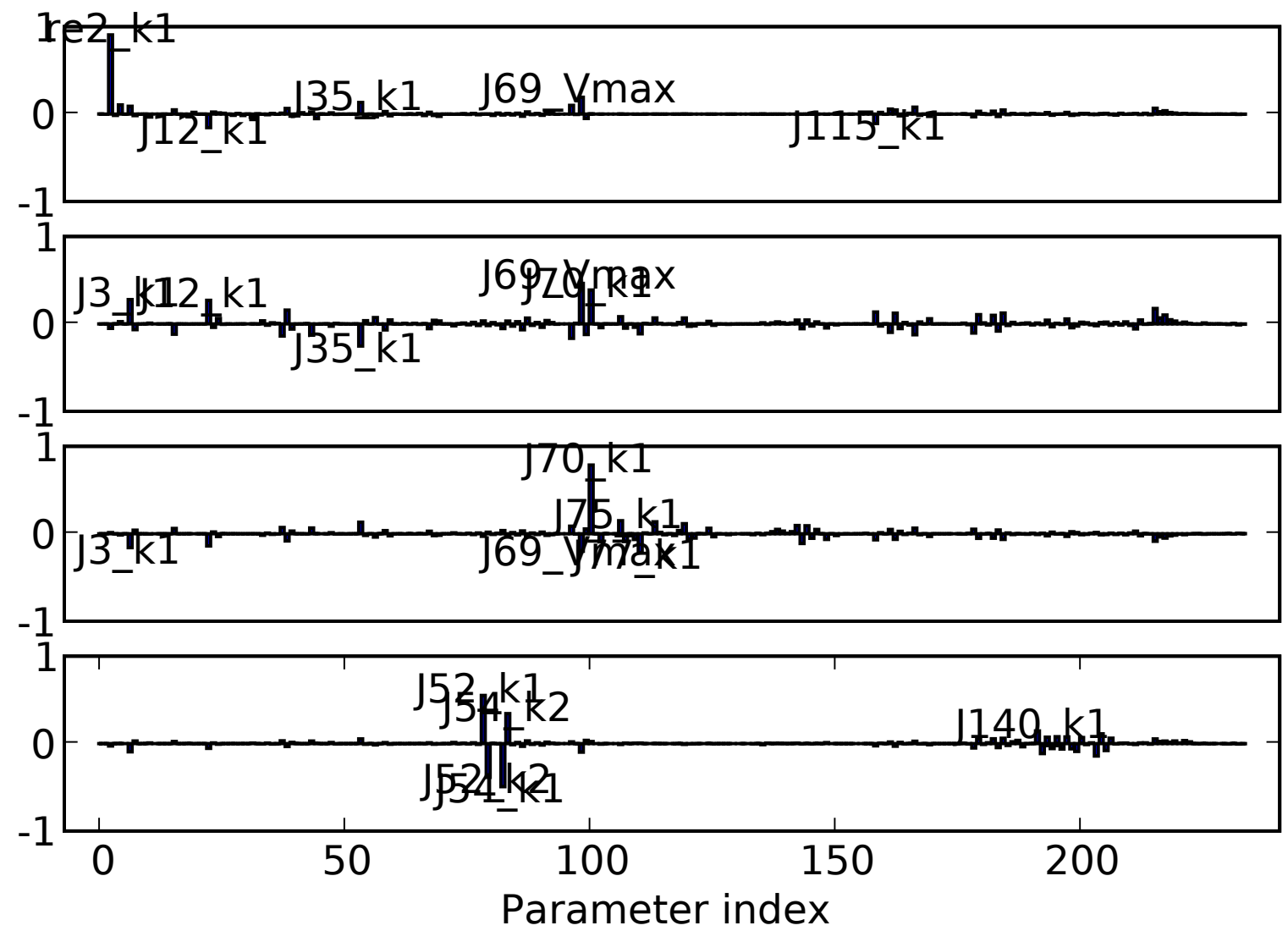

FIG. 17: Model (q): Sasagawa *et al.*'s model of rat growth-factor signaling [17].
