# Supplementary material for: Universally Sloppy Parameter Sensitivities in Systems Biology Models
Source: PLoS Comput Biol. 2007 Oct 5;3(10):e189. doi: 10.1371/journal.pcbi.0030189 (PMC2000971; doi:10.1371/journal.pcbi.0030189)
Supplement: Supplementary file 2 [file pcbi.0030189.sd003.pdf]

Universally Sloppy Parameter Sensitivities in Systems Biology Models
Supporting Text 2: Effect of Other Poorly Determined Parameters

Ryan N. Gutenkunst, Joshua J. Waterfall, Fergal P. Casey,
Kevin S. Brown, Christopher R. Myers, and James P. Sethna

The subfigures in Figure 1 correspond to Figure 4B from the main text, for missing the measurement of each of the 24 model parameters involved in our prediction. (Uncertainty in those parameters involved in the inhibited PI3K branch or in NGF-dependent C3G branch can have no effect on this prediction.) The blue regions are 95% confidence intervals given a single poorly determined parameter. For comparison, shown in red is the 95% confidence interval from precisely determining all parameters. For the exact role of each parameter in the model, see the original model paper [1].

---

[1] Brown KS, Hill CC, Calero GA, Myers CR, Lee KH, et al. (2004) The statistical mechanics of complex signaling networks: nerve growth factor signaling. Phys Biol 1:184–195.

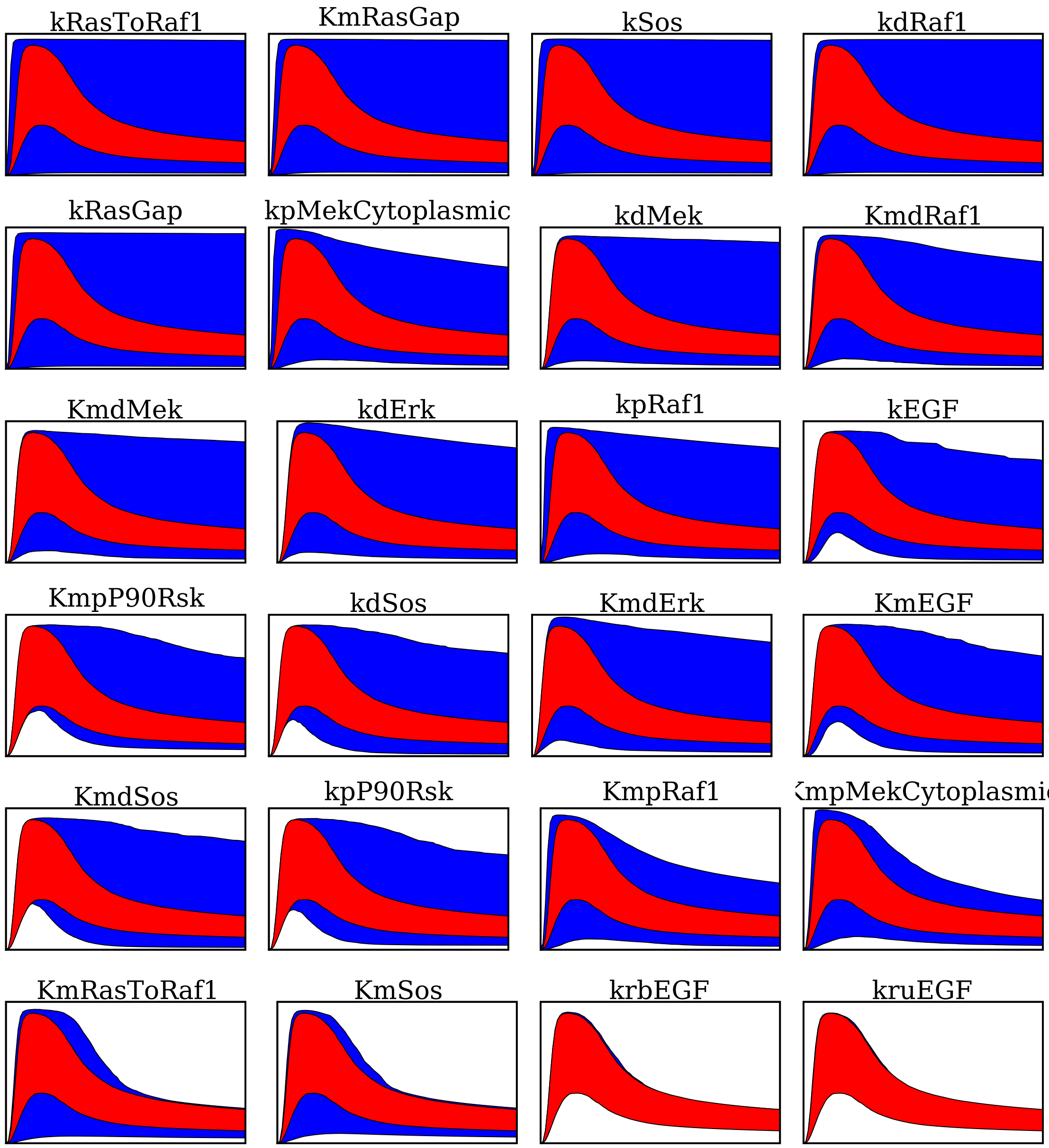

FIG. 1: Effect of other missing parameters on our example prediction.
