# Supplementary material for: Universally Sloppy Parameter Sensitivities in Systems Biology Models
Source: PLoS Comput Biol. 2007 Oct 5;3(10):e189. doi: 10.1371/journal.pcbi.0030189 (PMC2000971; doi:10.1371/journal.pcbi.0030189)
Supplement: Supplementary file 3 [file pcbi.0030189.sd004.pdf]

Universally Sloppy Parameter Sensitivities in Systems Biology Models
Supporting Text 3: Fragility of Other Predictions

Ryan N. Gutenkunst, Joshua J. Waterfall, Fergal P. Casey,
Kevin S. Brown, Christopher R. Myers, and James P. Sethna

Figures 1 and 2 show the 95% confidence intervals for two other example predictions, as in Figure 4B from the main text. The red intervals correspond to measuring all parameters to within plus or minus 25% (at 95% confidence). The blue intervals correspond to measuring all parameters as before, except for one, which is estimated to within a total range of 1000. The yellow intervals correspond to the collective parameter fit from Brown et al. [1].

Figure 1 shows a prediction of the activity of Ras given EGF stimulation in a wild-type cell. Missing a measurement of the rate constant for activation of p90 by Erk yielded the large blue interval.

Figure 1 shows a prediction of Mek activity given NGF stimulation of a wild-type cell, with the blue region corresponding to a missing measurement of the rate constant for the activation of B-Raf by Rap1. In this case the collective fit gave only an upper bound on the activity of Mek, so precisely measuring each individual parameter would yield a stronger prediction than the collective fit. (A measurement of zero Mek activity upon stimulation would be consistent with the model as constrained by the collective fit, but inconsistent the model as constrained by direct parameter measurements.) Nevertheless, the prediction with one missing parameter measurement remains much less informative than the collective fit.

[1] Brown KS, Hill CC, Calero GA, Myers CR, Lee KH, et al. (2004) The statistical mechanics of complex signaling networks: nerve growth factor signaling. Phys Biol 1:184–195.

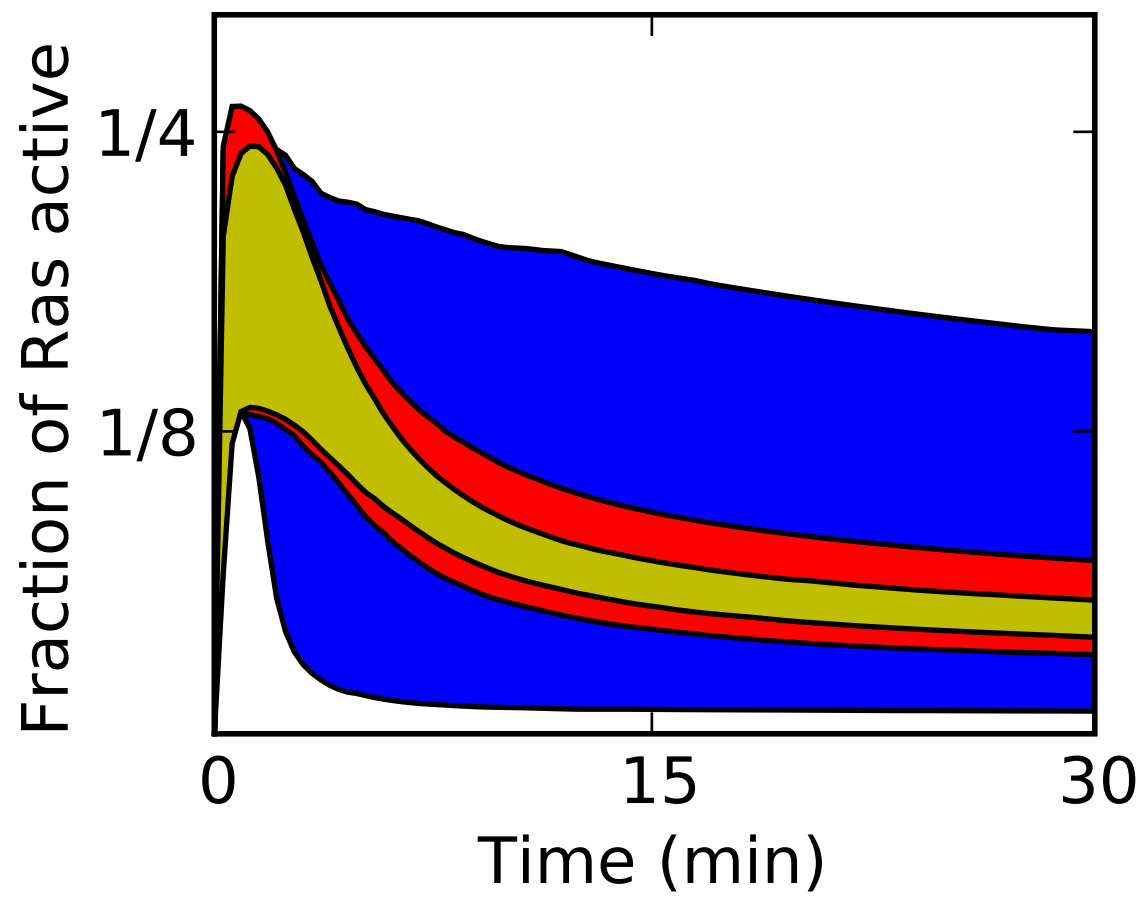

FIG. 1: Prediction uncertainties for Ras activity given EGF stimulation.

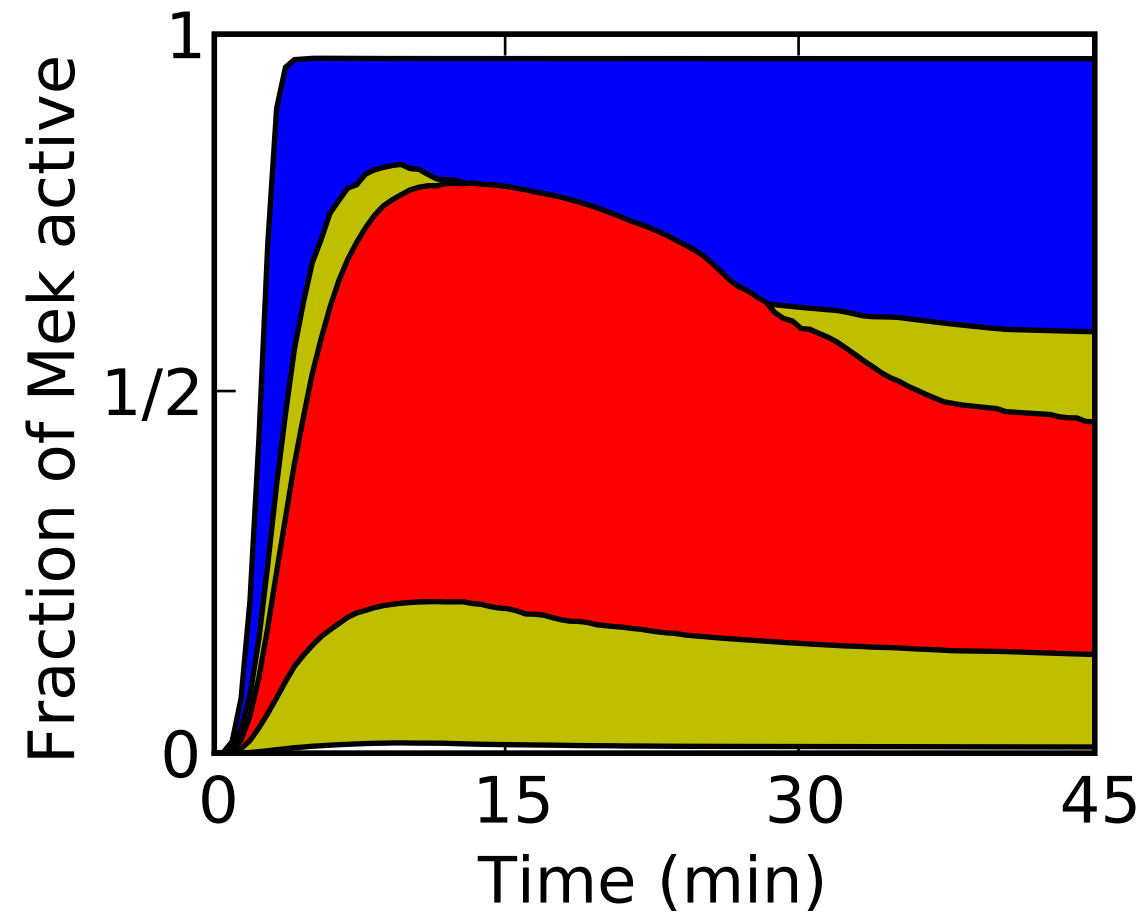

FIG. 2: Prediction uncertainties for Mek activity given NGF stimulation.
