# Supplementary material for: Universally Sloppy Parameter Sensitivities in Systems Biology Models
Source: PLoS Comput Biol. 2007 Oct 5;3(10):e189. doi: 10.1371/journal.pcbi.0030189 (PMC2000971; doi:10.1371/journal.pcbi.0030189)
Supplement: Supplementary file 4 [file pcbi.0030189.sd005.pdf]

Universally Sloppy Parameter Sensitivities in Systems Biology Models
Supporting Text 4: Rescaled Model of Brown et al.

Ryan N. Gutenkunst, Joshua J. Waterfall, Fergal P. Casey,
Kevin S. Brown, Christopher R. Myers, and James P. Sethna

To study the effect of time and concentration scales on sloppiness, we consider two modified versions of the model of Brown et al. [1].

In the first ('Rescaled'), we attempt to adjust concentration and time scales while maintaining fit quality. All concentrations are scaled to one, and all Michaelis constants are set to one. Additionally, the binding of EGF to its receptor is set to equilibrium, to remove one known sloppy mode. (NGF binding must be slow to fit the experimental data.) All rate constants are then re-optimized, adding an additional constraint on their total range. The resulting fit to the data has an approximately 50% higher cost. The eigenvalues of H^{χ^2} for this version of the model are shown in the central column of figure 1.

To fully remove the effects of time and concentration scales in the model, we set all non-zero initial conditions to 1 and all parameters to 1 ('All One'). The resulting eigenvalues of H^{χ^2} are plotted in the right column in figure 1.

Note that both adjusted models remain sloppy, with eigenvalues roughly evenly spaced over many decades.

[1] Brown KS, Hill CC, Calero GA, Myers CR, Lee KH, et al. (2004) The statistical mechanics of complex signaling networks: nerve growth factor signaling. Phys Biol 1:184–195.

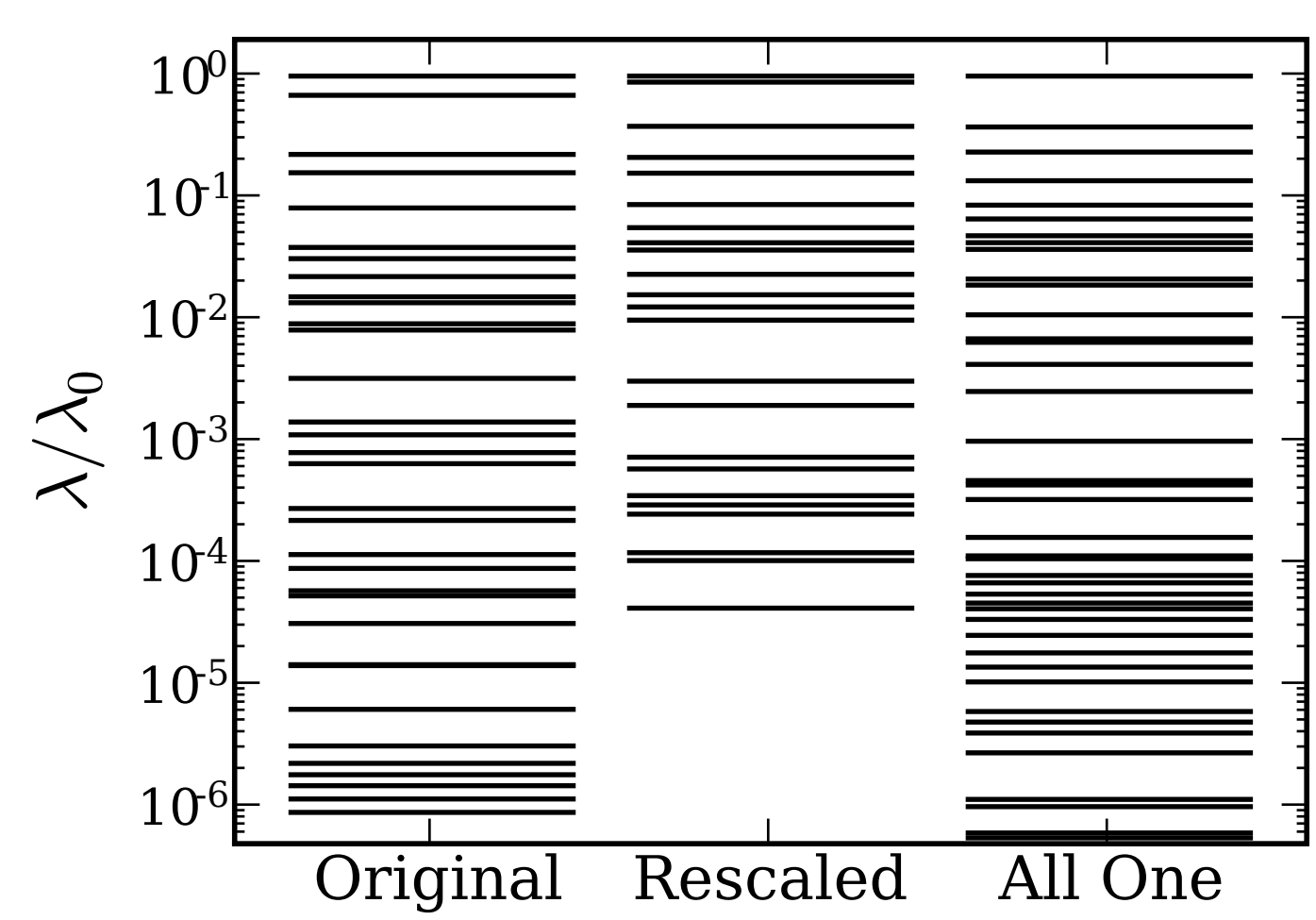

FIG. 1: Eigenvalue for rescaled Brown *et al.* models.
