# Supplementary material for: Universally Sloppy Parameter Sensitivities in Systems Biology Models
Source: PLoS Comput Biol. 2007 Oct 5;3(10):e189. doi: 10.1371/journal.pcbi.0030189 (PMC2000971; doi:10.1371/journal.pcbi.0030189)
Supplement: Supplementary file 5 [file pcbi.0030189.sd006.pdf]

Universally Sloppy Parameter Sensitivities in Systems Biology Models
Supporting Text 5: Sloppy Model Analysis of Brodersen et al. Binding Studies

Ryan N. Gutenkunst, Joshua J. Waterfall, Fergal P. Casey,
Kevin S. Brown, Christopher R. Myers, and James P. Sethna

Brodersen *et al.* [1] studied the equilibrium binding of salicylate to human serum albumin and of oxygen to hemoglobin. In both cases, they fit a binding model by least-squares to data consisting of ligand per protein versus free ligand concentration. They repeated the fitting procedure 30 times in each case, terminating each optimization once a parameter set was found that yielded an acceptable fit to the data within the experimental noise.

The resulting two collections of parameter sets are not statistically weighted ensembles like that we built to make predictions from the Brown *et al.* model. Nevertheless, Brodersen *et al.*'s collections of acceptable parameter sets likely approximate such statistical ensembles. Using principal component analysis to fit a multidimensional gaussian to each of Brodersen's parameter collections yields a Hessian matrix we can test for sloppiness.

Figure 1 shows eigenvalue spectra derived from Brodersen's acceptable parameter sets for the Albumin (Alb ens) and Hemoglobin (Heme ens) models. For comparison, the eigenvalues of the χ^2 Hessians for the two models are also shown (Alb chi^2 and Heme chi^2).

The sloppiness of both models is evident, using both our χ^2 measure of system behavior and with Brodersen's parameter set collection. In each case the eigenvalues span several decades roughly evenly.

---

[1] Brodersen R, Nielsen F, Christiansen JC, Andersen K (1987) Characterization of binding equilibrium data by a variety of fitted isotherms. Eur J Biochem 169:487–495.

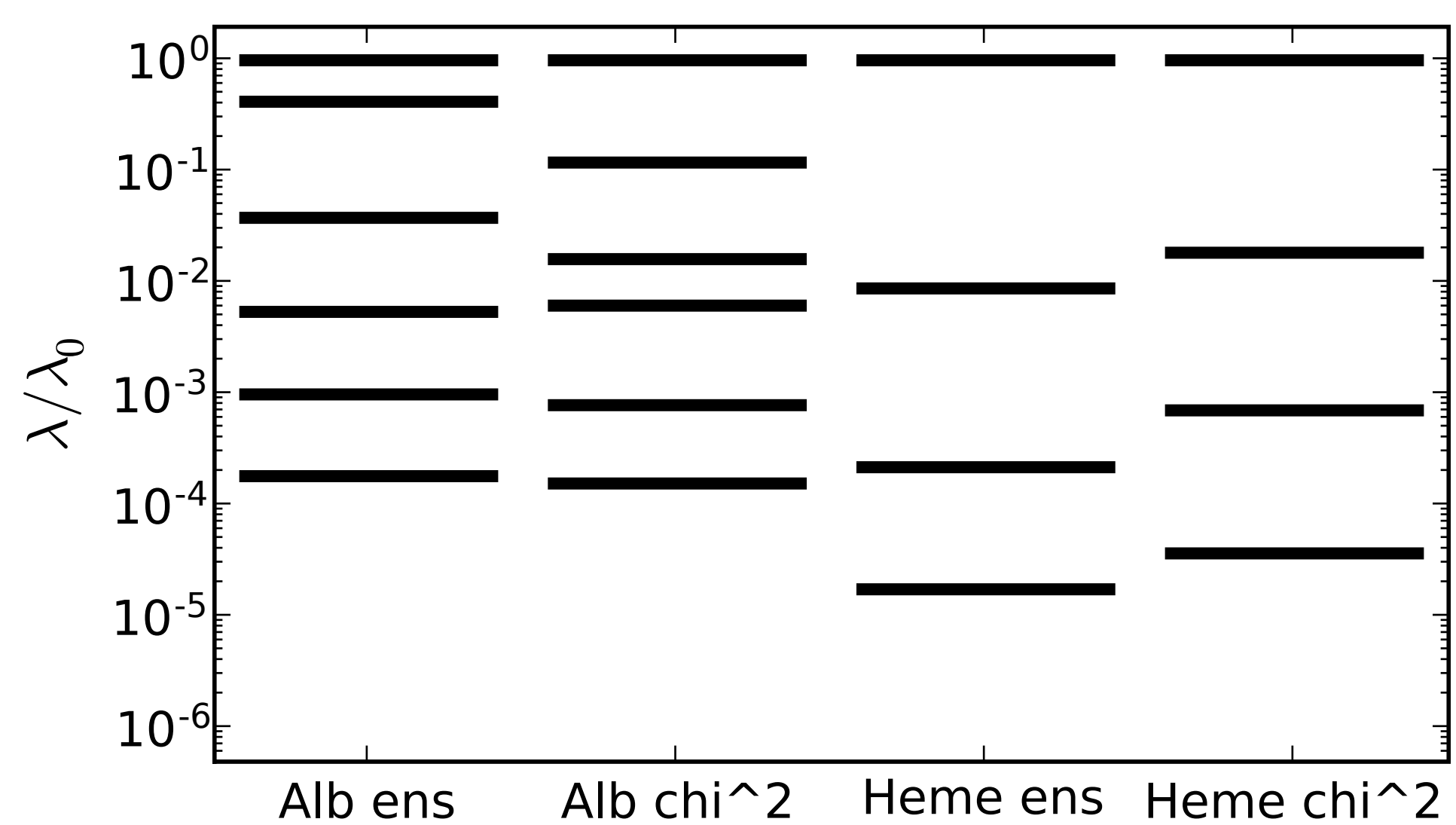

FIG. 1: Sloppy eigenvalues of Brodersen et al.'s models.
